# ‘Barriers to overcoming the barriers': A scoping review exploring 30 years of clinical supervision literature

**DOI:** 10.1111/jan.15283

**Published:** 2022-05-16

**Authors:** Roselyne Masamha, Lolita Alfred, Ruth Harris, Sally Bassett, Sarah Burden, Annette Gilmore

**Affiliations:** ^1^ Department of Psychological Health Well‐being and Social Work University of Hull Hull UK; ^2^ School of Health Sciences City University of London London UK; ^3^ Florence Nightingale Faculty of Nursing, Midwifery and Palliative Care King's College London London UK; ^4^ Faculty of Health and Life Sciences, Headington Campus Oxford Brookes University Oxford UK; ^5^ School of Health Leeds Beckett University Leeds UK; ^6^ Professional Record Standards Body London UK

**Keywords:** barriers, clinical supervision, critical reflection, facilitators, nurses, organizations, skills

## Abstract

**Aims/Questions:**

To explore the barriers and facilitators to nurses accessing clinical supervision; explore the barriers and facilitators to organizations implementing clinical supervision and capture what skills nurses require to facilitate clinical supervision.

**Design:**

Scoping review of peer‐reviewed research and grey literature.

**Data sources:**

CINAHL, Medline, PsychINFO and Scopus were searched for relevant papers published between 1990 and 2020. Google, Google Scholar, OpenGrey & EThOS were used to search for grey literature.

**Review Methods:**

PRISMA‐ScR guidelines were used during the literature review process. Eighty‐seven papers were included, and data were extracted from each paper using a standardized form. Data synthesis was undertaken using Seidel's analytical framework.

**Results:**

Five themes were identified: Definitions and Models, (Mis) Trust and the Language of Supervision, Alternative Parallel Forums and Support Mechanisms, Time and Cost and Skills required.

**Conclusion:**

Since its inception in the 1990s, clinical supervision has long been regarded as a supportive platform for nurses to reflect on and develop their practice. However, this review highlights that despite an awareness of the skills required for nurses to undertake clinical supervision, and the facilitators for nurses to access and organizations to implement clinical supervision, there have been persistent barriers to implementation. This review identifies these persistent factors as ‘*barriers to overcoming the barriers'* in the clinical supervision landscape. These require critical consideration to contribute towards moving clinical supervision forward in the spirit of its original intentions.

**Impact:**

This review progresses the debate on clinical supervision through critically analysing the barriers to overcoming the barriers. To this end, the review is designed to stimulate critical discussions amongst nurses in different clinical spaces and key stakeholders such as policy makers and regulatory bodies for the nursing profession.

## 1 INTRODUCTION

Clinical Supervision (CS) was first introduced to United Kingdom (UK) nursing practice over 30 years ago. At this time, CS was defined as a formal process of professional support and learning, enabling practitioners to develop knowledge and competence, assume responsibility for their own practice and enhance consumer protection and patient safety in clinical situations (Department of Health, 1993). This definition set the standard, at a national level, drawing on the seminal work of Butterworth and Faugier (1992) who set out this original purpose. It was developed in response to recognition of nurses' need for support and the continuous need for reflection on and development of their practice. The intention was for CS to be introduced to all nurses, however, while it gained traction in some fields of nursing practice (in particular, mental health), it has not been widely or consistently implemented in all nursing fields. Despite patchy and problematic implementation, the idea of CS continues to persist (Driscoll et al., 2019). Internationally, CS has been implemented in other countries, for example Australia (Sharrock et al., 2019), the United States of America and some countries within Europe (Cutcliffe & Lowe, 2005; Cutcliffe & Owen, 2017).

The potential for CS as a mechanism to support nurses in stressful situations, has recently received renewed attention as a result of the high levels of anxiety and post‐traumatic stress disorder (PTSD) experienced by nurses during the COVID‐19 pandemic (Couper et al., 2022). The Royal College of Nursing (RCN) (2021a) emphasized the importance of revisiting the fundamental aspect of supporting staff, as services begin to resume normal activities. The RCN (2021b) highlighted that ‘*effective and regular supervision must be in place to help identify and address issues of moral injury and strengthen patient safety*’ (online), through and beyond the pandemic. However, it is important to note here that although CS may contribute to staff support approaches, the use of CS in this way would be a deviation from its original purpose.

This paper presents a scoping review of CS literature over the last 30 years, with a specific focus on facilitators and barriers to access, from both individual and organizational perspectives. Furthermore, it identifies what the literature outlines as the skill set required by nurses to support CS implementation, consistency and continuity.

## 2 BACKGROUND

CS in the nursing discipline has been widely defined, discussed and debated. Differences and similarities in definitions are apparent within and between countries and fields of nursing practice (Colthart et al., 2018; Cross et al., 2010; Hyrkäs, 2005; Keegan, 2013; White & Winstanley, 2021; Williams et al., 2005; Wilson, 1999). There remains a call for an articulation of CS at the policy level, to enable individuals and organizations to work with a consolidated definition as a benchmark for a national context. This would avoid misappropriation of clinical supervision practice to suit organizations' own needs or political needs (Australian Clinical Supervision Association, 2015 cited in White & Winstanley, 2021). Though opinions remain divided, both in the UK as well as internationally, over whether CS for nurses should be made mandatory or not, professional codes and service regulators contend that CS contributes to professional development, quality care and supports the safe practice of the profession. Regulatory bodies such as the Care Quality Commission (CQC) in the UK, further identify the benefits of clinical supervision, linking it specifically to regulatory aspects of good governance and fitness to practice (CQC, 2013). However, this has not explicitly translated into the Nursing and Midwifery Council (NMC) Code of Practice (NMC, 2018) or the previous NMC (2010) version. For example, the NMC 2010 standards state, ‘They [nurses] must aim to improve their performance and enhance the safety and quality of care through evaluation, supervision and appraisal’ (p6). The NMC 2010 standards for competence further indicate, ‘They [nurses] must maintain their own personal and professional development, learning from experience, through supervision, feedback, reflection and evaluation’ (p7). The use of the term ‘supervision’ in isolation as opposed to ‘clinical supervision’ leaves it open to interpretation. The current NMC (2018) standards make even less reference to supervision. Interestingly, within the proficiencies for Mental Health Nursing practice, CS is specifically emphasized.

The following selected examples demonstrate that the focus of publications on CS has shifted over time, from initial articles outlining definitions, models and potential benefits of CS (Butterworth, 1994; Fowler, 1996; Severinsson & Hummelvoll, 2001; Wilson, 1999) , to literature addressing how CS is implemented for nurses in practice, with implementation strategies divided into issues of ‘method’ and ‘practicality’ (Berg & Hallberg, 1999; Scanlon & Weir, 1997). This was followed by the need to evaluate its effectiveness (Buus et al., 2010; Cutcliffe, 1997; Gonge & Buus, 2010) and the preparation of practitioners for their roles as supervisor and supervisee (Ashmore & Carver, 2000; Cutcliffe et al., 1998). The shift then progressed to critically examining and analysing emerging issues in the implementation of CS (Cleary & Freeman, 2005; Grant & Townend, 2007; White & Winstanley, 2010). Then more recently, an examination of conceptual issues within CS and nursing (Banks et al., 2013; Howard & Eddy‐Imishue, 2020; Pollock et al., 2017; Puffett & Perkins, 2017).

The literature refers to models of CS in two different ways, first in relation to group CS and individual CS also known as ‘type or format of clinical supervision’. Second, models of CS are presented in relation to the conceptual frameworks guiding the delivery of CS—including for example the stages of the CS process, functions of CS, roles of supervisor and supervisee, an example being Proctor's Model (Sloan & Watson, 2002). Proctor's Model of CS developed in 1986 which like other models encapsulates the original purpose of CS, is the most cited as used in practice, structured through three key areas. **Normative**: promoting and complying with policies and procedures, developing standards and contributing to clinical audit; **Formative**: developing skills and evidence‐based practice; **Restorative**: enabling practitioners to better understand and manage the emotional burden of nursing practice (White & Winstanley, 2009a).

Various models of CS are proposed in the literature (Butterworth et al., 1997; Hyrkäs, 2005; Palsson et al., 1996), but there is no universal agreement on the correct model of clinical supervision for nursing. The idea of having more than one model is seen as a benefit, acknowledging that different clinical situations may require slightly different considerations, therefore more models allow nurses to use the most appropriate one for their specific needs (Fowler, 1996). How models are understood and challenges to their application in practice, are a recurrent feature across the literature. CS seminal authors such as Butterworth, Faugier and Burnard made early attempts to clarify CS models and their application in practice. However, these attempts were still viewed as confusing and not well articulated (Boggs, 1999; Duke, 1999).

While there is a significant amount of literature on CS, its expected impact on supporting and developing nurses has not been realized and implementation efforts have stalled. The literature explores facilitators of CS; barriers to its access and implementation, as well as the skills nurses require to facilitate CS. However, despite this research, the root causes accounting for the persistent challenges in implementation receive little attention. Therefore, this review progresses longstanding discussions on CS and provides a critical analysis of its value as a support mechanism for nurses to reflect on and enhance their practice. The review moves away from familiar recurring debates to adopt a more targeted approach, focusing on the reasons why CS has not been more widely implemented despite what is known about existing barriers and facilitators.

## 3 THE REVIEW

CS literature is extensive and complex, therefore adopting a scoping review approach enabled mapping of the literature in line with the review aims to provide an overview of concepts, evidence and research in the field (Pollock et al., 2021). The guidance on scoping reviews by Peters et al. (2020) was used. This ensured the review maintained integrity and robustness in the key elements of question formulation; inclusion and exclusion criteria articulation; a replicable search strategy with a decision flowchart and data extraction. Taking the scoping approach allowed for more responsiveness to the data that emerged iteratively.

### 3.1 Aim

The overarching aim of the review was to explore the extent, range and nature of evidence on clinical supervision, focusing specifically on the following three research questions:

- What are the barriers and facilitators to nurses accessing clinical supervision?
- What are the barriers and facilitators to organizations implementing clinical supervision for nurses?
- What skills do nurses require to facilitate clinical supervision?

### 3.2 Search methods

A systematic search of existing literature on clinical supervision was undertaken on four major electronic databases in November 2020, namely‐ CINAHL, MEDLINE, PsychInfo and Scopus. The databases were scanned using key search terms (and their permutations), as well as Boolean operators “OR” and “AND” to yield literature that was relevant to answering the review questions: The search terms used were as follows: (“Clinical Supervis*” **OR** “Reflective Supervis*” **OR** “Restorative supervis*” **OR** “Nurse supervis*” **OR** “Supervisory role*” **OR** “Peer support” **OR** “Continuing Professional Development”) **AND** (Nurs* **OR** Midwi* **OR** “Allied Health Professional*”) **AND** (barrier* **OR** obstacle* **OR** difficult* **OR** challenge* **OR** issue* **OR** problem* **OR** limitation* **OR** facilitat* **OR** enable* **OR** lever* **OR** promot* **OR** support* **OR** strengthen*). Acknowledging that there is a wealth of literature and resources on clinical supervision that might not be published in standard academic databases and journals, the search was extended to include Grey Literature—sourced from Google, Google Scholar, OpenGrey & the EThOS thesis/dissertation database. Extending the search in this way aligns with what the literature recommends (Centre for Reviews and Dissemination, 2009).

Inclusion and exclusion criteria were used to set the parameters for the scoping review (Patino & Ferreira, 2018). Table 1 lists the inclusion and exclusion criteria used to select papers that address the review aims. Both peer‐reviewed and non‐peer‐reviewed papers (such as commentaries and discussion papers) were included to capture the scope of understanding of CS. Papers written in English only were included (for practicality), however, it is acknowledged that some valuable resources written in other languages may have been missed as a result. The search terms included other professions such as allied health professionals and midwives to enable the initial search results to yield a broader range of literature. This was in recognition that there may be clinical supervision studies conducted with mixed professional groups that include nursing. However, as this scoping review is mainly interested in CS related to the nursing profession, any mixed professional group papers (identified at title, abstract and full‐text screening) that did not isolate results for nurses were consequently excluded.

**TABLE 1:** Inclusion & exclusion criteria

Inclusions	Exclusions
Empirical studies (quantitative, qualitative, mixed methods papers and literature reviews)	Papers that focus solely on other professional groups (midwifery, allied health professionals)
Other empirical work such as Theses	Papers not written in English
Editorials	
Commentaries	
Discussion papers	
Opinion pieces	
Nursing profession (including all fields—adult/general, learning disabilities, mental health and child nursing)	
Papers exploring mixed professional groups (midwifery, nursing, allied health professionals) if they clearly outline results in relation to nursing.	
Peer‐reviewed and non‐peer‐reviewed papers	
Papers published from January 1990 to November 2020	

### 3.3 Quality appraisal

In line with scoping review methodology presented by Peters et al. (2020), a quality appraisal of the included papers was not conducted, as the intention was to provide a scope of the literature on CS with emphasis on barriers, facilitators and skills of clinical supervision. Furthermore, as the review included grey literature such as discussion papers and opinion pieces, these may be regarded as lower quality evidence if using traditional standards of critical appraisal. Nevertheless, these were valuable sources of information that add depth of understanding of the scope of literature aligned with the review aims.

### 3.4 Data extraction and synthesis

To summarize and synthesize the key areas across the included papers, data from each paper were extracted and placed onto a matrix outlining author name/s, year of publication, paper aim/s, sample size, country/setting, type of study/article and nursing field of practice. The data extracted also included (as relevant) the barriers and facilitators to nurses accessing CS, barriers and facilitators to organizations implementing CS, and an outline of the skills nurses require to facilitate CS (see Tables S2, S3 & S4 in Supplementary File 2). Due to some papers being discussion pieces or commentaries, information such as sample size, country and nursing discipline was not always applicable—and this is reflected in the results tables. The data extraction of included papers was undertaken by all co‐authors (RM, LA, RH, SBa, SB, AG) with discussions and agreement on the paper synthesis and theme development.

**FIGURE 1:**
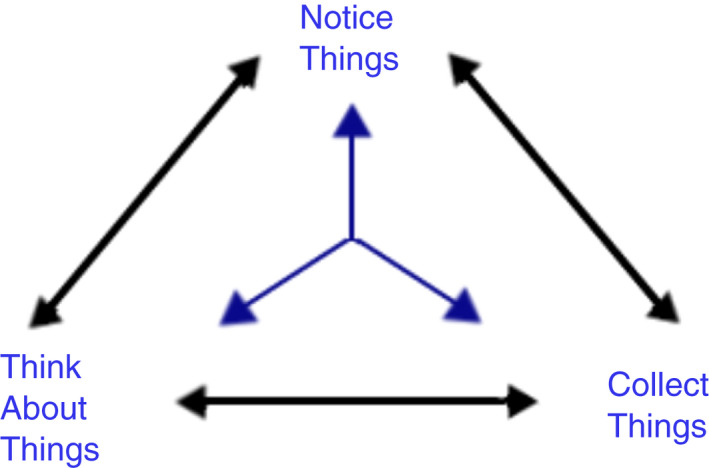
Analysis process (adapted from Seidel, 1998)

Data synthesis in relation to facilitators, barriers and skills was guided by an adaptation of Seidel (1998) model. This is made up of three main elements of Noticing, Collecting and Thinking about things (see Figure 1). This approach to synthesis was selected recognizing that the process of reviewing the CS literature was not linear, but cyclical. Adopting this model to the synthesis process enabled the application of a systematic structure to a complex analysis with high volumes of data. Through reading the papers repeatedly, themes were **noticed**. The themes emerging through the reading were **collected** and reflected on (**thinking about things**) using wider literature on CS. In this way, the elements in the CS data across the papers mirror the three elements identified in Seidel's, 1998 model. Data synthesis was undertaken by RM & LA and through discussion with RH, AG, SBa & SB the emerging themes were refined. Results were initially presented to demonstrate the papers which contributed evidence to answer the three research questions posed in Section 3.1: This was followed by the overarching thematic analysis exploring the factors influencing the implementation of CS.

## 4 RESULTS

### 4.1 Search outcomes

**FIGURE 2:**
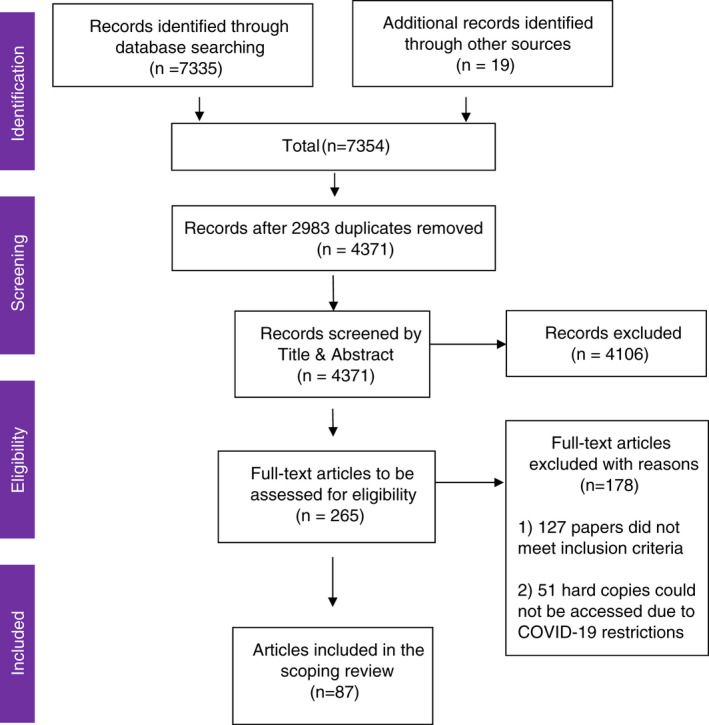
Decision flowchart (adapted from Tricco et al., 2018)

The database search yielded 7335 records, and a further 19 records were found through Google Scholar and EThOS. After eliminating duplicates, two authors (RM & LA) independently blind screened (according to title and abstract) the remaining 4371 papers using Rayyan, an online collaborative literature review tool. This process eliminated 4106 papers which did not meet the inclusion criteria and any conflicts at this stage were resolved through discussion. This left 265 papers for full‐text screening. The full‐text copies of 51 records (that were not available electronically), could not be obtained due to COVID‐19 restrictions preventing access to the locations where hard copies of these were held. Two authors (RH & LA) screened the titles and abstracts of the 51 unobtainable papers, the majority appeared to be commentaries, discussion papers and opinion pieces (based on their abstracts explicitly identifying this, or their lack of details on methodology and methods that would indicate the expected formatting of empirical, peer‐reviewed publications). See Supplementary File 1 for a list of the 51 papers. Of the 214 remaining records that were read in full, a further 127 were eliminated for not meeting the inclusion criteria. The remaining 87 papers were included in the scoping review. The screening decision flowchart is presented in Figure 2 using the adapted PRISMA extension for Scoping Reviews (PRISMA‐ScR) (Tricco et al., 2018).

### 4.2 Study characteristics

In total, 87 papers were included in the review. The papers were published between 1995 and 2020 and focused on the barriers and/or facilitators to nurses accessing CS, organizational barriers to implementing CS and the range of skills that nurses require to facilitate effective CS. Most articles that stated country of origin presented research undertaken in the UK (*n* = 31), then research studies from a range of other countries, Australia (*n* = 10), Denmark (*n* = 6), New Zealand (*n* = 3), Finland (*n* = 2), Sweden (*n* = 2), Norway (*n* = 1) and South Africa (*n* = 1). Country of origin was not specified or was unclear for 31 articles, many of which were commentary or discussion pieces. From the data available, only one study was conducted outside Global North. However, the English language inclusion criteria may have resulted in some non‐English language papers from other countries being excluded.

Fifty‐nine of the 87 included papers contributed evidence of the barriers and /or facilitators to nurses accessing CS. These papers are reported in detail in the supplementary material (Table S2 Supplementary File 2: and Table 2). Twenty‐one papers were discussion or opinion papers and 38 were empirical research (including quantitative, qualitative, mixed methods and literature reviews). Nearly two‐thirds (*n* = 36) of the papers were published in the last 16 years, which highlights the rise in the number of studies conducted and papers discussing barriers and interventions that might enhance nurses' engagement with or access to CS. These papers related to all the main nursing fields of practice; Learning Disability, Mental Health, Child and Adult/General, however, nearly one‐third (*n* = 19) of the papers explored the barriers and facilitators specifically within mental health or psychiatric nursing settings.

**TABLE 2:** Themes

	Theme 1: definitions & models	Theme 2: alternative parallel forums and support mechanisms	Theme 3: (Mis) trust & the language of supervision	Theme 4: time and cost	Theme 5: skills required
Ainsworth, 2000				✓	
Blackford & Street, 1998	✓			✓	
Brunero & Lamont, 2012				✓	
Bryant, 2010				✓	
Burrow, 1995			✓	✓	
Butterworth et al., 2008				✓	
Bush, 2005	✓		✓	✓	✓
Buus et al., 2010	✓				
Buus et al., 2013			✓		
Buus et al., 2011				✓	
Buus et al., 2018		✓		✓	
Cairns, 1998	✓		✓		
Castille, 1996				✓	
Chambers & Long, 1995			✓		✓
Cheater & Hale, 2001		✓	✓	✓	✓
Chilvers & Ramsey, 2009				✓	✓
Cleary & Freeman, 2005	✓	✓			
Cleary & Freeman, 2006			✓	✓	
Cleary et al., 2010				✓	
Clough, 2003	✓		✓	✓	
Colthart et al., 2018	✓				
Cotton, 2001			✓		
Cross et al., 2010	✓		✓	✓	
Cross et al., 2012				✓	
Cutcliffe et al., 1998			✓		
Cutcliffe & Proctor, 1998					✓
Darley, 2001	✓				
Dillon, 2014			✓		
Edwards et al., 2005				✓	
Fowler, 2013a, ^a^					
Fowler, 2013b, ^a^					
Fowler, 2013c				✓	
Freshwater et al., 2003			✓	✓	
Gonge & Buus 2010^a^					
Gonge & Buus, 2016, ^a^				✓	
Grant & Townend, 2007			✓		
Gray, 2001			✓		
Hancox et al., 2004				✓	✓
Harvey et al., 2020					✓
Hadfield, 2000				✓	
Howard & Eddy‐Imishue, 2020		✓	✓		
Howatson‐Jones, 2003		✓			
Hughes & Morcom, 1998			✓	✓	
Jenkins et al., 2000				✓	
Johansson et al., 2006			✓		
Jones‐Berry, 2018				✓	
Keegan, 2013	✓		✓	✓	
Kelly et al., 2001			✓		
Koivu, HyrkÄS, & Saarinen, 2011, ^a^					
Koivu, Saarinen, & Hyrkas, 2011, ^a^					
Lister & Crisp, 2005			✓	✓	
Long et al., 2014			✓	✓	
Lowry, 1998			✓	✓	
Lynch & Happell, 2008			✓		
Lyon, 1998, ^a^					
Malin, 2000			✓		
McCarron et al., 2017				✓	
O'Connell et al., 2011				✓	
Olsson et al., 1998, ^a^					
Puffett & Perkins, 2017			✓	✓	✓
Rice et al., 2007	✓				✓
Robinson, 2005			✓	✓	
Severinsson & Hallberg, 1996			✓		✓
Severinsson, 1996					✓
Sexton‐Bradshaw, 1999			✓		✓
Sines & McNally, 2007			✓		
Sloan 1998					✓
Sloan, 1999a					✓
Sloan, 1999b					✓
Sloan, 2008					✓
Smith, 2001			✓	✓	
Spence et al., 2002			✓	✓	
Stacey et al., 2020					✓
Stevenson & Jackson, 2000		✓	✓		
Stevenson, 2005			✓		
Temane et al., 2014					✓
Tobias, 2016				✓	
Turner et al., 2005, ^a^					
Webb, 1997				✓	
White & Winstanley, 2006				✓	
White & Winstanley, 2009a				✓	
White & Winstanley2009b				✓	
White & Winstanley, 2010		✓	✓	✓	
Williams et al., 2005	✓				
Williams & Irvine, 2009				✓	
Wilson, 1999					✓
Wright, 2012				✓	

^a^ 8 papers did not contribute directly to the themes that emerged from our analysis; however, they did capture and contribute to understanding the barriers or facilitators to nurses accessing CS, the barriers or facilitators to organizations implementing clinical supervision or the articulation of the skillset required by nurses to undertake CS.

Fifty‐four of the 87 included papers contributed evidence which focused on organizational barriers to implementing CS (Table S3 Supplementary File 2, and Table 2). Fifteen papers were discussion or opinion pieces and 39 were empirical research (including qualitative, quantitative and mixed methods designs). These papers represented Learning Disability, Mental Health, Child and Adult/General nursing and the organizational barriers were found to be similar across these nursing fields of practice. The majority (*n* = 28) of the papers were published between 2000 and 2010 which illustrates how the debate had moved on from discussing models to exploring the organizational responsibilities, threats and opportunities for providing and facilitating CS.

Nineteen of the 87 included papers contributed evidence that focused on the range of skills that nurses require to facilitate effective CS (Table S4, Supplementary File 2 and Table 2). They were all empirical research including qualitative, quantitative evaluation designs and literature reviews. The papers were mainly written in relation to the mental health or psychiatric nursing field (*n* = 7) and adult/general nursing (*n* = 6); five did not specify the field of nursing. Although other papers may have mentioned the importance of training and support, it was these 19 that went into specific details that either clearly identified the types of skills and/or analysed the benefits of such skills in a clinical supervisor; and/or explored ways to enhance training and formal recognition of CS skills. The papers that identified the skills nurses required to facilitate CS were predominantly from the UK.

### 4.3 Themes

Five key themes were identified in the evidence from the 87 included papers which are explained below. These were as follows:

- Definitions and models.
- Alternative parallel forums and support mechanisms.
- (Mis) Trust and the language of supervision.
- Time and cost.
- Skills required for CS.

#### 4.3.1 Definitions and models

The theme of definitions and models explicitly focuses on the aspect of differences in definitions and models, illustrating that there remains concern over the absence of a universally acceptable definition of CS and the ongoing struggle with the concept, within and across the nursing profession. The issue of numerous definitions of CS is cited as a hindrance to progressing the implementation of CS, with literature emphasizing the need for a consistent definition. This perception is evident in most papers as indicated in Tables S2 and S3 (Supplementary File 2). However, Antrobus (1997) points out that the purpose of nursing and the knowledge base required to practice nursing are debates that should be a continuous feature in healthcare, influenced by the evolving nature of health and the dynamic political and social climate in which nurses practice.

The struggles of professional identity that have long troubled the nursing profession, have driven emphasis on defining a unique epistemology in ways that tend to present nursing knowledge as securely anchored on fixed standardized understandings. This perception that equates fluidity to a lack of substance, is in our view—*a barrier to overcoming the barrier* of inconsistent definitions. In this context, no singular and standardized definition of CS can explain what it is that nurses need. Clarity about the purpose and underlying principles allow for a focus on designing CS to meet needs in the most appropriate way rather than seeking or trying to secure a fixed definition.

#### 4.3.2 Alternative parallel forums as support mechanisms

The theme of alternative parallel forums as support mechanisms was presented as legitimate opportunities for staff to receive support to reflect on and develop their practice much like CS. Schwartz Rounds, Handovers, Post‐incident Debriefs, Serious Untoward Incident Analysis (SUIA), Case formulations, multi‐disciplinary team meetings, right through to conversations with family and friends have all been cited in the literature as favoured alternatives to CS (Banks et al., 2013; Cleary & Freeman, 2005; Taylor et al., 2018; White & Winstanley, 2010). A range of papers that focused on engaging with nurses who did not participate in CS suggested nurses' preferences for alternative forums and a perception amongst nurses of more satisfactory support from these alternatives. For example, Cleary and Freeman (2005), highlighted that although nurses were aware of the advantages of CS, many preferred ad hoc coping methods such as informal sharing and eliciting the support of trusted colleagues than more formal approaches. Informal support with one's peers was seen to be more responsive to the clinical realities of everyday work as, generally, colleagues were available and accessible. The emergence and availability of alternative parallel forums as support mechanisms were perceived as a barrier to nurses participating in CS as it was originally designed and thought to negatively influence nurses' commitment to CS.

It would seem counterproductive to disrupt an effective alternative forum of support to insist on CS which may not, as a replacement, be seen to offer equal support. This prompts a position that views alternative forums through the lens of broadening nurse support mechanisms. It is important to note that this is not suggesting that CS should not be encouraged or that it should be substituted where it is working well, but acknowledging that other forums may be equally or even perhaps more supportive to the development of nursing practice and patient outcomes. Alternative parallel forums as a form of support, have in common with CS, a professional's ability to reflect and learn in a supportive context. This is with an acknowledgement that the ad‐hoc nature and lack of formal structure which makes these alternative forums more attractive to nurses, is simultaneously problematic in terms of consistency, continuity and measurement of impact. It is further noted that the objectives, value and usage of these was not fully explored or measured by the studies included in this scoping review and this requires further exploration and measurement of impact.

The complex dual nature of alternative forums should be considered as a platform to explore whether the emphasis should be; to push for a collective understanding of CS to unify understanding across contexts, or to reframe it. Allowing for varied understandings and conceptualizations of CS and therefore choice of access may result in more widespread implementation, moving the debate on. The focus is on ensuring nurses are supported as opposed to what that support is labelled as. Provided alternative parallel forums are aligned to the purpose of developing practice, the view of alternative parallel forums as competitive rather than complementary became a *barrier to overcoming the barrier of* Alternative Parallel Forums & support mechanisms.

The issue of alternative sources of support is not a simple issue of preference, it is laden with more complex issues around nurse relationships and nursing culture. A selected quote from a participant in research by White and Winstanley (2010) highlights several entanglements surrounding the active decision to rely on alternative support. This quote is selected for its demonstration of the myriad of issues and indication of the complex entanglements that surround and lie beneath, decisions to disengage with CS; it also draws together similar issues raised by other nurses (see Table S2—Supplementary File 2). The text in bold (our emphasis), brings into sharp focus the multiple aspects at play that govern engagement decisions. “**I don't buy into it** [CS]. **Its stuff that they do** and **I chose not to participate in it**. I just **can't see any point in it**. I've **learnt how to deal with the crap we put up with day to day** and **it's just a culture** I feel is **never going to change**, because it's **just the way it is**. It's **just the way we are**. I've been working for [organization] for nearly [number] years. I've seen a change from what we used to do, to what we are doing now, and you **learn to compartmentalize it**. But, yeah, I just *don't feel the need* to participate in a forum like that, I guess. From my personal point of view, I think *it's* **flogging a dead horse.** No, I **don't need to talk about work**. If I ever need to talk to someone, I will **go home and debrief with my partner**. We **do it over the coffee table** as well…” (White & Winstanley, 2010, p. 692). This quote highlights the multiple layers across which barriers manifest and the messy realities of nursing work, nursing practice and nursing spaces. Tackling these requires thinking with complexity and simultaneous forms of address. It is interesting that the alternatives identified are ones that either involve other professionals in addition to nurses or do not have nurses in them at all (family support). This observation also illustrates the theme of (Mis) Trust and the language of CS amongst nurses, as an aspect that undermines the value nurses place on CS.

#### 4.3.3 (Mis)Trust and the language of supervision

The theme of (mis)trust and the language of supervision represents the association of CS as a tool for reprimand and discipline, which has its roots in the NMC's prescribing of CS as a response to issues of poor practice (Fowler, 1996). CS has since struggled to rid itself of this stigma and CS was regularly cited in the literature as being a form of performance management and surveillance rather than support (Dillon, 2014; Lister & Crisp, 2005; Puffett & Perkins, 2017). Furthermore, the conflation of managerial and clinical supervision combined with its facilitation and control by managers has continued to reinforce the surveillance perception, where CS may be used to compensate for poor management practice (Gray, 2001) and performance management (White & Winstanley, 2021).

This association of CS with performance management makes it a challenge to position CS as a positive form of learning designed to support nurses, something separate from the assessment and monitoring of their performance. Several authors have drawn attention to the pedagogical value, reflective opportunity and value of cognitive development with little success (Severinsson,1996). Some attempts to reconceptualize CS considering the challenges identified have additionally introduced a new term that does not include the word ‘supervision’ such as Egalitarian Consultation Meetings by Stevenson and Jackson (2000). Despite efforts to reassure nurses that CS is a positive aspect of professional development, a continued sense of mistrust has been the *barrier to overcoming the barrier* of the language of supervision.

#### 4.3.4 Time and cost

The theme of time and cost encapsulated the tension between the recognition of the importance of time and cost of successful access and implementation of CS, against commitment to action this recognition. Further complicated by considerable variation in the frequency and time spent on supervision during each session (Sexton‐Bradshaw, 1999).

White and Winstanley (2009b) articulate the false economy of time and cost as reasons for not supporting CS. They highlight that those staff who probably need CS most, are those least likely to receive it and/or facilitate it for other people. Furthermore, there is a stronger argument for CS in areas where staff are busier due to the demands of clinical settings in which they work. The impact on retention and the cost of subsequent recruitment, stress‐related sick pay and other indirect financial costs such as organizational reputation outweigh the cost of supporting CS and building time for it at a strategic level. In an earlier paper, White and Winstanley (2006), undertake a cost–benefit analysis while simultaneously considering other readily accepted care elements such as handover. They argue that, CS being built into the working day and regarded as legitimate work, would be no different from other activities such as handover; universally accepted and considered necessary even as they remove clinical staff from direct patient care. White and Winstanley (2006) argue for CS to be an explicit national standard for nursing practice, with consequences of its non‐occurrence equated to negligence. CS not being viewed as ‘real work’ is a well‐emphasized barrier within the literature (see Tables S2 & S3—Supplementary File 2).

The absence of a mandate on CS has led to CS not being readily recognized consistently as an integral part of professional activity and professional competency within the context of work. Hadfield (2000) recommended formal linkage to Post Registration Education and Practice (PREP) as a means of getting the status, time and resources needed for CS. There may be merit in revisiting this earlier recommendation, now in the context of revalidation, as a mechanism that can be used to give CS legitimacy. Not affording CS a legitimate work status within the profession translates into a *barrier to overcoming the barrier* of time and cost.

#### 4.3.5 Skills required for CS

Training in CS is recommended, and studies have shown this helps (See Table S4—Supplementary File 2). Training was identified as central to developing skills that would enable more effective clinical supervision facilitation (Chilvers & Ramsey, 2009; Puffett & Perkins, 2017). Furthermore, clinical supervisors who had received clinical supervision training evaluated better than those who had not undertaken any training at all. However, the review highlighted that the training and education for nurses to facilitate CS varied across the literature, from hours to days, to full modules and courses. There were also variations in the facilitator requirements from experienced CS supervisors in practice to university lecturers.

What staff valued as skills in CS supervisors also differed, from counselling skills to generic people handling skills. There were some particularly interesting papers that suggested a specific skill set that is more therapeutic in nature, for example a clinical supervisor requires the skills to respond to and contain the distress or emotional disclosure shared by supervisees (Stacey et al., 2020). Wilson (1999) identified counselling skills would be essential while Severinsson and Hallberg (1996) identified the ability to show understanding, genuine feelings and ‘confirming’, to validate the supervisees' and the ability to be patient and sensitive to situations ‘in the air’. Some papers emphasized training and skills in relation to the field of practice or client group supported, as opposed to skills for the facilitation of clinical supervision per se. For example some skills were based on the supervisor being an expert in that clinical area (Sloan, 1999a). Communication and good listening skills were the most cited necessary skills (Chilvers & Ramsey, 2009; Puffett & Perkins, 2017; Sloan, 1999a; Sloan, 1999b; Temane et al., 2014; Wilson, 1999).

## 5 DISCUSSION

CS remains a key recommendation for supporting nurses to reflect on and develop their practice. This scoping review explored the three questions which centred on the barriers and facilitators to nurses accessing clinical supervision; barriers and facilitators to organizations implementing clinical supervision for nurses and the skills nurses require to facilitate clinical supervision. The review identified 87 papers (summarized in the supplementary files) which answered these questions. The five themes explored in the Results section exposed and articulated the current impasses to CS being embedded in routine clinical practice. These themes re‐captured the well‐acknowledged barriers to the implementation of and access to clinical supervision as identified by authors such as Howard and Eddy‐Imishue (2020) in the literature. Hence, bringing into sharp focus the fact that despite the knowledge of the facilitators and efforts to upskill nurses in CS, there remains a persistent set of barriers to effective implementation. The familiarity of these themes suggests that within the nursing profession, we may have come to confuse the regularity with which we mention CS to be indicative of progress while neglecting the tensions that come with increased emphasis. Therefore, this discussion is deliberately designed to evoke and provoke critical thought around the themes identified in this review.

In relation to the facilitators to nurses accessing and organizations implementing CS, it is worth noting that what is presented as facilitators in some of the literature, are the perceived benefits of CS, with an erroneous assumption that knowledge of the advantages alone would translate into facilitators. There appears to be a lack of clarity between aspirations and outcomes of CS, this potentially limits its successful implementation and the quality of evaluations undertaken.

In relation to the barriers to nurses accessing and organizations implementing CS, the review highlights some of the tensions around CS that have historically challenged and still currently challenge the profession (Banks et al., 2013) individual nurses as well as the organizations in which they work. The persistent and repetitive nature of the barriers to CS becomes a central and overarching dynamic that this review critically examines as the *barriers to overcoming the barriers*. While this review sheds light on these issues, it acknowledges that given the broad fields of nursing practice as well as the time span across which these same barriers were evident (albeit with nuanced particularities), warrants further critical exploration through research and discussion in clinical spaces.

In relation to the skills necessary for nurses to facilitate effective CS, while this is seen as central (Puffett & Perkins, 2017), it is difficult to determine, from this review, clarity and consistency of the specific skills and knowledge that nurses require. This difficulty is largely due to the variation in the models of CS, the nursing setting, discipline and experience of the nurses. The wide variations in acceptable skill and knowledge base of supervisors to inform their implementation of CS also present difficulties for the evaluation of its effectiveness. However, considering the existing and persistent barriers undermining the successful implementation of CS; there is an argument to be made that knowledge may not necessarily be the problem, rather, a commitment to action.

The review indicates that the challenges of implementing CS are beyond surface issues of practicality highlighted in the early debates. Ongoing analysis points to much deeper‐seated tensions within the nursing profession, as well as the seemingly established assumptions about the purpose and effectiveness of CS itself. It is important to understand the complex contextual factors that influence how CS is perceived, introduced and managed. The current scoping review proposes that over the last 30 years, a set of assumptions or ideologies of CS have developed about its perceived acceptance, application and value by the profession. This may, to some degree, hinder objective reevaluation of the purpose and role that CS plays in developing effective contemporary nursing practice. CS as intended, embodies the opportunity to learn from events that enable individual professional growth along a proficiency continuum (Benner, 1982), whereby expertise or professional mastery can be achieved.

The review has a few limitations. First, the absence of an agreed set of terms to define/refer to CS as well as the multiplicity of terms used to describe CS complicated the identification of relevant papers. Nevertheless, all efforts were made to incorporate as many alternative terms as possible to increase the chances of locating the most relevant papers. Second, a total of 51 papers (that were unavailable electronically) could also not be located as hard copies for full‐text screening due to COVID‐19 access restrictions. However, a list of these papers is offered as supplementary material for transparency (see Supplementary File 1). Third, the review did not explore CS in the context of pre‐registration nursing preparation as this was beyond the scope of this review, however it is acknowledged that this may be an area for future reviews to focus on. Finally, papers written in other languages were excluded for practicality, however, it is acknowledged that some valuable resources written in other languages may have been missed as a result. Despite the above limitations, however this review provides a valuable synthesis of the wide range of CS literature over a 30‐year timeframe and makes an original contribution towards understanding why CS barriers seem to persist, and why the acknowledged facilitating factors or even the knowledge/skills of how to implement or undertake CS have not influenced wider and more consistent implementation.

Based on the findings of this review, recommendations* are that future research and practice needs to be solution focussed in relation to:

1. Factors underlying the persistent barriers as opposed to restating what they are across different settings and fields of nursing.
2. Missing knowledge areas such as support mechanisms for agency nurses and internationally recruited nurses, and the provision of culturally specific and effective approaches to supporting diverse nursing staff. The literature in the current scoping review did not attend to these areas explicitly, however this may be an area for future exploration because a significant proportion of the nursing workforce is employed through nursing agencies and international recruitment.
3. Acknowledging alternative parallel forums as complementary to CS rather than being seen as competing against it.
4. Critical reflections on the resistance to interprofessional CS given that the concept of CS is not unique to nursing, and because the alternative parallel forums from which nurses report benefit are themselves interprofessional.
5. Policy and regulation should be responsive to all the above recommendations.
6. The need as a profession to examine and debate our identity as critical reflective learners, for whom reflective practice is expected; access and support is an organizational obligation, leaving only the form CS takes as a personal choice.
7. Preregistration nursing preparation was beyond the scope of this review; however, it is acknowledged that this may be an area for future reviews to focus on.

*These recommendations are not suggesting order of priority

## 6 CONCLUSION

CS has long been regarded as a supportive platform for nurses to reflect on their practice. Although there are some clear enablers to CS; since its inception in the 1990s, it has been mired with barriers to implementation. What is presented in the literature challenges views about widely accepted perceived benefits of CS. Additionally, knowledge about the advantages of CS resulting in successful integration into practice, is not reflective of the reality for all nurses across the United Kingdom. The current scoping review raises some important considerations regarding whether CS currently serves its intended purpose, particularly, whether an emotional attachment to the idea of CS hinders critical questioning of its effectiveness and value in contemporary nursing practice. There needs to be a recognition of the possible dissonance between a strong desire for/ belief in CS, against the baggage of poor CS experiences, limited evidence of its successful implementation, uptake and impact. What is needed now is for the profession to lay bare the barriers to overcoming the barriers of CS as identified in this review. Furthermore, to engage in an honest, dispassionate critical re‐examination of the approaches required to facilitate professional growth and support within nursing.

## AUTHOR CONTRIBUTIONS

**Roselyne Masamha** led on developing the search strategy, screening of papers, data extraction, data synthesis/interpretation, theme development, and writing the article; **Lolita Alfred** led on developing the search strategy, screening of papers, data extraction, data synthesis/interpretation, theme development, and writing the article; **Ruth Harris** led the conception of the study, and contributed to writing the review proposal, screening of papers, data extraction, data synthesis and interpretation and reviewed drafts of the article; **Sally Bassett** led the conception of the study, and contributed to writing the review proposal, screening of papers, data extraction, data synthesis and interpretation and reviewed drafts of the article; **Sarah Burden** led the conception of the study, contributed to writing the review proposal, screening of papers, data extraction, data synthesis and interpretation and reviewed drafts of the article; **Annette Gilmore** led the conception of the study, contributed to writing the review proposal, screening of papers, data extraction, data synthesis and interpretation and reviewed drafts of the article.

## Supporting information


Appendix S1

Appendix S2

## Data Availability

Data available in article supplementary material
