# Supplementary material for: ‘Barriers to overcoming the barriers': A scoping review exploring 30 years of clinical supervision literature
Source: J Adv Nurs. 2022 May 16;78(9):2678–92. doi: 10.1111/jan.15283 (PMC9546137; doi:10.1111/jan.15283)
Supplement: Supplementary file 1 — Appendix S1 [file JAN-78-2678-s002.docx]

**SUPPLEMENTARY FILE 1**

**List of unobtainable full text papers**

1. Bainbridge D., Butterworth C; Mills J. (2001). Clinical Supervision. Occupational Health, 53(3): 16-17.
2. Baxter, T. (1995). Clinical supervision in action: with care of the elderly mentally ill. Source: Professional Update 3(6): 45-46.
3. Bishop, V. 1996. Editorial... clinical supervision. NT Research, Mar-Apr; 1(2): 91-91.
4. Bishop, V. (1996). Focus. Clinical supervision. *NT Research*, *1*(2), 94–95.
5. Bishop, V. (1998). Clinical supervision: what is going on? Results of a questionnaire including commentary by Butterworth T. *NT Research*, *3*(2), 141–151. <https://doi.org/10.1177/174498719800300212>
6. Bishop, V. (1998). Clinical supervision: what’s going on? Results of a questionnaire... this paper is an edited version of one appearing in NT Research 1998; 3:2, 141-150. *Nursing Times*, *94*(18), 50–53.
7. Bond, M and Holland, S. (2011) Skills of Clinical Supervision for Nurses: A Practical Guide for Supervisees, Clinical Supervisors and Managers. Second Edition. Crit Care Nurse 1 October 2011; 31 (5): 78. doi:<https://ebookcentral.proquest.com/lib/rcn/detail.action?docID=714187>
8. Brocklehurst N. (1997). Clinical supervision in nursing homes. *Nursing Times*, *93*(12), 48–49.
9. Buus, N and Gonge , H(2012) Participation and benefits of clinical supervision of psychiatric nursing staff Klinisk Sygepleje - Volume 26, Issue 4, p35-48
10. Catmur S. (1995). Clinical supervision in mental health nursing. *Mental Health Nursing*, *15*(1), 24–25.
11. Cole A. (2002). Someone to watch over you supervision. *Nursing Times*, *98*(23), 22–25.
12. Collins J. (1995). Clinical supervision: a blessing or a curse? *Occupational Health*, *47*(11), 387–388.
13. Cowe F, & Wilkes C. (1998). Clinical supervision for specialist nurses. *Professional Nurse*, *13*(5), 284–287.
14. Dixon A, & Bramwell R. (2001). Neonatal nurses’ attitudes to clinical supervision: results of a survey. Journal of neonatal nursing volume 7 p20-24.
15. Duarri W, & Kendrick K. (1999). Update. Implementing clinical supervision. *Professional Nurse*, *14*(12), 849–852.
16. Farquharson A, Trotter G, & Nimmo S. (1998). Clinical supervision and practice nurses. *Nursing Times*, *94*(25), 52–53.
17. Faugier J. (1994). Thin on the ground. *Nursing Times*, *90*(20), 64–65.
18. Fowler J. (1995). Nurses’ perceptions of the elements of good supervision. *Nursing Times*, *91*(22), 33–37.
19. Freshwater D, Walsh L, & Storey L. (2002). Establishing clinical supervision in prison health care. *Foundation of Nursing Studies Newsletter*, 2–3.
20. Fulton J, & Oliver D. (2001). Clinical supervision: a psychodynamic approach. *Research for Nursing Practice*, *3*(1), 7p.
21. Gallinagh R, & Campbell L. (1999). Clinical supervision in nursing: an overview. *Nursing Review*, *17*(3), 52–56.
22. Gallinagh R, & Campbell L. (2000). Education for clinical supervision. *Nursing Review*, *18*(1), 11–12.
23. Greenwood J. (2001). Getting a grip on clinical supervision. *Journal of the Australasian Rehabilitation Nurses’ Association*, *4*(1), 11–15.
24. Hunter M. (2001). The provision of clinical supervision within a managerial hierarchy. *Assignment*, *7*(2), 12–17.
25. Jones, A (2001) Possible influences on clinical supervision. Nursing Standard - Volume 16, Issue 1, pp. 38-42
26. Kennedy, W (2006) Professional supervision to enhance nursing practice. Vision (11749784) - Volume 14, Issue 2, pp. 3-6
27. Kolade 2005 Nurses perception of clinical supervision in Nigeria. West African Journal of Nursing - Volume 16, Issue 1, pp. 11-19
28. Kopp, P (2001) "Fit for practice. 6.6: Clinical supervision [continuing education credit]". Nursing times, volume 97 issue 27, p. 47-49
29. Kwai, k (2002) Clinical supervision: a literature review. Whitireia Nursing Journal - Volume 0, Issue 9, pp. 37-41.
30. Lees, C (2000) The name is not the thing in supervision...Nursing Times' Open Learning series on clinical supervision. Nursing Times - Volume 96, Issue 6, pp. 25-25
31. Lucas, S and Jones, A (2000) Learning curve. NT open learning: clinical supervision -- part 8: implementation problems. Nursing Times, volume 96 issue 11, p49-52
32. Macinnes, A (1996) Don't scrimp on clinical supervision. Health Visitor, volume 69 issue 11, p476
33. Martin, J, Simpson, F, Marchant C, Baden, S, and Munday E (1996) Clinical supervision. Practice Nurse - Volume 11, Issue 3, pp. 159-162
34. McCallion, H.; Baxter, T. (1995) Clinical supervision. Nursing Management - UK - Volume 1, Issue 9, pp. 20-21
35. McHale, A (2002) Clinical supervision in community nursing practice. N2N: Nurse2Nurse - Volume 2, Issue 6, pp. 12-12
36. Ohlsson, E and Arvidsson, B (2005) The nurses' conception of how clinical supervision can promote their mental health. Nordic Journal of Nursing Research & Clinical Studies / Vård i Norden - Volume 25, Issue 2, pp. 32-35
37. Ooijen, E.V. (1994). Whipping up a storm: Will the nursing profession use clinical supervision in a punitive, controlling way? Els van Ooijen hopes not and makes the case for a culture of openness. *Nursing Standard, 9*, 48-50.
38. O'Riordan, B. 2002. Why nurses choose not to undertake clinical supervision--the findings from one ICU. *Nursing In Critical Care.* 7 (2), pp. 59-66.
39. Oxley, P (1995) Clinical supervision in community psychiatric nursing. Mental Health Nursing - Volume 15, Issue 6, pp. 15-17
40. Parsons, M (2001) Clinical supervision. Journal of the Australasian Rehabilitation Nurses' Association. Volume 4, issue 4, p15-17
41. Porter, N (1996) The value of networking: introducing clinical supervision into nursing homes. Elderly Care, volume 8, issue 5, p35
42. Sams, D 1996 Clinical supervision: an oasis for practice. Community Health Nursing, volume 1 issue 2 p87-91
43. Sheerin, F Clinical supervision: is it on the way? Nursing Review - Volume 17, Issue 4, pp. 103-106
44. Street, S (2008) Clinical supervision. Dissector, volume 36, issue 1, p6
45. Stringer EW. A study of supervised practice from the perspective of the supervisor and the supervised. Insight. 2003 Jul-Sep;28(3):70-4. PMID: 14596139
46. Teasdale, K (2000) Practical approaches to clinical supervision. Professional Nurse volume 15, issue 9, p579-582
47. Thomas, A (2005) Clinical supervision is good for nurses' health...Nursing Times. Vol 101, issue 4, p16
48. Waterworth, S, Pillitteri, L and Swift F (1997) Clinical supervision: empowerment in practice... within a haematology nursing development unit. Nursing Management vol 3 issue 9 p14-16
49. Wilkin p, Bowers, L and Monk (1997) Clinical supervision: managing the resistance. Nursing Times, volume 93, issue 8 p48-49
50. Wolsey, P., & Leach, L. (1997). Clinical supervision: a hornet's nest. Nursing times, 93 44, 24-7
51. Wright SG, Salmon D, Maddock J, Watts J, Sandford L, Thomas D, Crowder J, Hopkins V. Clinical supervision: making it happen. *Elder Care.* 1998 Oct-Nov;10(5):10-4. PMID: 9866496.
