# Supplementary material for: ‘Barriers to overcoming the barriers': A scoping review exploring 30 years of clinical supervision literature
Source: J Adv Nurs. 2022 May 16;78(9):2678–92. doi: 10.1111/jan.15283 (PMC9546137; doi:10.1111/jan.15283)
Supplement: Supplementary file 2 — Appendix S2 [file JAN-78-2678-s001.docx]

**SUPPLEMENTARY FILE 2**

**Table 2: Literature Review Question 1 - Nurses Barriers and Facilitators to Accessing Clinical Supervision**

| Author/s | Year Published | Country / Region | Nursing Field /Setting | Number of Participants | Aim | Barriers | Facilitators | Type of Paper |
| --- | --- | --- | --- | --- | --- | --- | --- | --- |
| Blackford & Street | 1998 | Australia | Paediatric cardiac nurses | 8 Post graduate paediatric cardiac nurses | To evaluate the model’s suitability for professional development in nursing as an educational tool in post graduate nursing programmes | Time | Ability to build and maintain trust and a quiet place to meet, method/model to be agreed | Research |
| Burrow | 1995 | N/A | N/A | N/A | Explores whether clinical supervision is clinical development or management control | Insufficient differentiation between managerial & clinical supervision, perception of clinical supervision as management control & scrutiny, purist clinical supervision approach not reflective of the broadening role of the nurse (not solely practitioner focussed), policing of clinical supervision - to prove cost-effectiveness & organisational benefit, having to account for supervisees whose practice may fall short of standards |  | Opinion Piece |
| Bush | 2005 | N/A | Nurses- generic | N/A | Discusses the barriers, reasons for the barriers, and overcoming them | Confused with performance review; viewed as being about criticism and attributing blame; it is imposed rather than voluntary; reluctance to disclose personal issues; fear of losing autonomy; feels like self-indulgence for some and clinical supervision seen as having problematic definition | Requires assured confidentiality; better communication; clear, simple definitions conveying benefits / relevance, nurses need to feel as if they own the process | Discussion Paper |
| Buus et al., | 2013 | Danish study | Psychiatric Nursing- wards | Ward A- 5, 4, & 3 participants @ three test sessions. Ward B- 4, 3, & 4 participants | Explain the development, implementation, and adjustment of the manual for the intervention, describe the content and structure of the final educational intervention, and reflect on six key-issues in relation to implementing the manual. | Keeping participants engaged between sessions was a challenge; summaries of their reflection sent by email was not sufficient, where the clinical supervision relationship was authoritative participants were hesitant to voice their own thoughts and opinions. | Sensitivity of the group supervisor to potential issues of shame and fear of self-disclosure among participants is an important facilitator | Research (Implementation Study) |
| Buus et al., | 2010 | Denmark | Psychiatric Nurses | 9 General psychiatric wards at a university hospital in Denmark, purposive sample of 22 informants: (15 frequent participants & 7 infrequent participants) | Investigate psychiatric hospital nurses’ accounts of participating in group-based clinical supervision and to see what light psychosocial conceptions of group dynamic clinical supervision might shed on contradictory experiences. | Shift work resulted in variation in group size, supervision scheduled on days off or during an adjacent shift, poor staffing levels and continually shifting workloads made it difficult for staff to leave their work tasks to participate in supervision. Group clinical supervision sessions could demonstrate the limited knowledge of some supervisees which could cause discomfort e.g., being “professionally stripped in front of the group” or being “overwhelmed by an unanticipated emotional response”, staff felt clinical supervision had a short-term effect outside of sessions because there were low numbers of staff who actually participated in the group sessions | Receiving assistance in coping with the psychological burden | Research (Interviews) |
| Buus et al., | 2018 | Denmark | Mental Health Nursing | 24 | Resistance to group clinical supervision | Pre-existing conflicts, lack of trust, having to use your own time, additional commute to work, no perceived benefits, disclosure of personal & professional uncertainty, resistance to being analysed, finding benefit in other parallel processes e.g. peer support, clinical reviews - seen as providing more supportive environments & relevance to clinical practice, years of experience viewed as providing ability to critically reflect on their own practices without collegial intervention or formalized supervision. Feelings that no one will listen so clinical supervision is pointless |  | Research (Interviews) |
| Buus et al., | 2011 | Danish study | Mental Health Nursing | 22 | To explore psychiatric hospital nursing staff reflections on participating in clinical supervision | Cost of participation - interrupting days off, perceptions of limited clinical value & impact | Nurses adopting an understanding of their professional practice as something continually under threat of becoming an undesirable routine and requiring an external supervisor to help them reframe clinical problems. Opportunity to articulate 'higher truth' about their professional work, participation in clinical supervision viewed as enhancing professional identity, clinical supervision viewed as an indication of 'caring' through being willing to challenge routines & be open to learning | Research (Interviews) |
| Cairns | 1998 | N/A | N/A | N/A | Discusses how practice nurses can make a considerable contribution to the development and delivery of primary care services. | Little evidence of clinical supervision within primary care. It could be argued that this is due to a lack of understanding of what the concept is about, where it comes from and why it is needed. Term 'supervisor' is traditionally associated with industry and commerce and is linked conceptually to a controlling authority, this may be a barrier to its effective implementation. practice development is dominated by medical, nursing and managerial hierarchies! With so many hierarchies already struggling to control and direct can the simple process of reflection with a peer ever carry enough power to influence practice. | Remove practitioner suspicion and develop useable local models otherwise it won’t be used by those who need this most. Mix disciplines to create more capacity for clinical supervision | Discussion Paper |
| Castille | 1996 | UK | Accident and Emergency (General Nursing) | N/A | To describe a model for clinical supervision which was specifically designed to support and enhance accident and emergency nursing practice. | Cost implications in terms of training as well as the loss of staff from the workplace during the supervision sessions | Group discussion facilitated by an experienced A&E nurse using a selection of recent patient record cards to help staff reflect on practice. Peer group review is linked to weekly teaching sessions. informal, non-threatening setting. Training is imperative to reduce resistance and provide an understanding of what supervision is - and what it is not | Discussion Paper |
| Chambers & Long | 1995 | N/A | N/A | N/A | To address some of the scholarly themes and concepts involved in the experience and management of supportive clinical supervision and to stimulate future dialogue and further discussion |  | Create an environment where supervisees to disclose aspects of their work without fear of reproach or disciplinary proceedings. Valuation and unconditional positive regard must be actively and intuitively ‘felt’ by both the supervisor and the supervisee. supervisor and supervisee must have commitment to the supervisory process. The supervisor is not hierarchically linked to the supervisee. Having a distraction free environment. Giving supervisee the freedom to explore and examine his or her clinical competence in a non-punitive way | Discussion Paper |
| Cheater & Hale | 2001 | England | Practice nurses | Unclear - (states all practice nurses in Leicestershire) | Evaluation of clinical supervision scheme. Assess the level of uptake of clinical supervision by practice nurses; identify factors which hindered/facilitated uptake; evaluate how far clinical supervision had influenced i) quality of clinical care ii) organisation of care iii) professional development | Good peer support already available through other platforms, practice nurses too experienced to benefit, not convinced of the benefit | Linking supervision to educational accreditation systems, or more explicitly to UKCC PREP requirements were suggestions for providing additional incentives for practice nurses to nurses to become involved (UKCC 1995) | Evaluation |
| Chilvers and Ramsey | 2009 | England | Hospice | Not specified | To explore practicalities of setting up clinical supervision for nurses, healthcare assistants & community associate practitioners across 3 hospice sites | Time Commitment | Supervisors actively involved in programme design | Literature Review |
| Cleary & Freeman | 2006 | N/A | Mental Health Nursing | N/A | To outline pragmatic issues concerning clinical supervision and advocate for facility-specific professional development and support processes that incorporate succession planning opportunities | Suspicion, tokenism, resistance, and interpersonal difficulties. Where external facilitators have been used, but they are often unable to commit to more than a few sessions and thus the benefits are questionable and the experience of clinical supervision negative. Work rostering can make it difficult to prioritise time for clinical supervision. Places free of distraction and interruption are next to impossible to find, lack of available rooms. Scepticism about confidentiality. Informal and social networks seem to preclude confidentiality, perpetuating vulnerability for those most exposed. The supervisor is often a colleague or manager who may develop particular agendas or interpretations of situations based on what they have heard during the session and inadvertently breach confidentiality by expressing strong prejudices. confusing terminology. | Having a supervisor outside of the supervisee’s clinical area can result in positive experiences from their participation in clinical supervision. Ensuring that the group is not hierarchical and group norms are established. Because of limited time, process of supervision must be clinically meaningful, user friendly, and relevant. | Discussion Paper |
| Cleary & Freeman | 2005 | Not specified | Acute Mental Health Nursing | 10 individual interviews with nurses who had been observed and 5 discussion groups | To explore nurses’ perceptions of professional attitudes and support to better understand the cultural realities of clinical supervision in acute inpatient mental health settings | One-to-one clinical supervision was considered impossible due to unit constraints. Rostering for the 24/7 inpatient service was a challenge to organising group clinical supervision. Difficulty creating space and time in the busy working day | Preferred ad-hoc coping methods such as informal sharing and eliciting the support of trusted colleagues than more formal approaches | Research (Ethnographic Study) |
| Cleary et al., | 2010 | N/A | N/A | N/A | To discuss the purpose of clinical supervision, the importance of its planned implementation, and its role relative to other in situ support strategies. | Time taken away from patients, added clinical burdens borne by colleagues, the challenges of managing workloads, and restricted time limits in high pressure settings mean that nurses question the benefits. In group supervision. Shift work and complex clinical work limits access to clinical supervision. | Supervisor and supervisee must be appropriately matched. not be involved in a current (or potential) management position with the supervisee. clinical supervision should be voluntary. Supervisor should be available, easily accessible, and chosen by the supervisee | Discussion Paper |
| Clough | 2003 | N/A | N/A | N/A | To demonstrate the value of clinical supervision for community nurses | Name 'supervision' suggestive of being judged, criticised or assessed by supervisor. Lack of time. Question regarding confidentiality. Rural issues, especially the problem of travelling time for practitioners working over large areas who are isolated both geographically and professionally from colleagues. Interpersonal problems or lack of motivation | Group supervision format was a facilitator for overcoming barriers and team integration | Discussion Paper |
| Colthart et al., | 2018 | N/A | N/A | N/A | To provide practical steps for implementing clinical supervision. |  | Prompts for supervisees on what they want out of supervision | Discussion Paper |
| Cross et al., | 2010 | Australia | General Nursing (acute medical ward) | 6 ANUMs participated in 15 group sessions in total and 4 took part in the focus group. | To implement and evaluate group clinical supervision for associate nurse unit managers (ANUMs) in a busy medical ward. | Questions whether word ‘supervision’ acceptable, or more problematic as signifying ‘oversight’ and ‘watching’. | Need for dedicated time | Evaluation (Pilot Study) |
| Cross et al., | 2012 | Australia | ICU Outreach- General Nursing | 2 | To explore the importance and perceived benefits of clinical supervision during the transition to the ICU outreach role, from the perspective of the two Outreach Nurses and the Supervisor. |  | Having a specific time set aside for clinical supervision was welcomed and they ensured that they preserved it within the confines of a hectic workplace | Research (Case Study) |
| Cutcliffe et al | 1998 | N/A | N/A | N/A | Discussion paper on ethical issues in clinical supervision (Part 2) | The existence of a tradition and culture that discourage the public expression of emotion. The perception of clinical supervision as yet another management monitoring tool. The perception of supervision as a form of personal therapy. A continuing lack of clarity regarding the purpose of supervision. Fear of potential ethical dilemmas arising for supervisors |  | Discussion Paper (part of a series) |
| Cutcliffe and Proctor | 1998 | UK | N/A | N/A | Discussion paper focussing on the issues of training in clinical supervision (Part 1) |  | train nurses to become supervisees not supervisors | Discussion Paper (part of a series) |
| Dillon | 2014 | New Zealand | N/A | N/A | Discussion paper acknowledging reciprocal vulnerability | Misinformed perspectives, views that nursing is paid for work, it is noble work, superhuman powers to not be affected by difficult work, seeking support is a sign of weakness, mistrust, defensiveness, horizontal and vertical bullying | Reciprocal vulnerability, emotional intelligence to be able to appreciate colleague emotional needs, supervisors also receiving support, valuing clinical supervision as an integral part of practice | Discussion Paper |
| Edwards et al., | 2005 | England | Community Mental Health Nursing | 260 | To identify factors that may influence the effectiveness of clinical supervision for community mental health nurses | Trust rapport, personal issues, time, importance of supervision |  | Research |
| Fowler | 2013a | N/A | N/A | N/A | Discussion paper focusing on advancing practice through clinical supervision |  | Openness to self-reflection; personal commitment; willingness to grow | Discussion Paper (part of a series) |
| Fowler | 2013b | N/A | N/A | N/A | Discussion paper focusing on advancing practice through clinical supervision |  | Find the right mentor; putting oneself and needs first; making long term plans | Discussion Paper (part of a series) |
| Freshwater et al., | 2003 | England | Prisons | 35 | To establish and evaluate a strategy for the effective implementation of clinical supervision within a group of prisons and subsequently to make recommendations for good practice, identifying real and perceived barriers | Inability to take responsibility, staff relations, apathy and lack of motivation. |  | Research (Mixed Methods) |
| Gonge & Buus | 2016 | Danish study | Psychiatric Nursing | 115 | To test whether a meta- practice intervention could increase psychiatric nurses’ clinical supervision participation and effectiveness of existing supervision practices, and to explore organizational constraints to implementation of these strengthened practices | The lower participation and effectiveness of clinical supervision was due to largescale organisation change which impacted the intervention group. diminishing experience of social support from colleagues was associated with reduced participation in clinical supervision, while b) additional quantitative demands were associated with staff reporting difficulties finding time for supervision |  | Research (Longitudinal Study) |
| Gonge & Buus | 2010 | Danish study | Psychiatric Nursing | 239 | To investigate how often psychiatric nursing staff participate in clinical supervision and any possible associations among individual and workplace factors that influence participation | Staff experiencing high cognitive demands was less likely to participate in clinical supervision | Experiencing social support from colleagues was associated with a higher participation rate | Research (Survey) |
| Grant & Townend | 2007 | UK | Mental Health Nursing | N/A | Paper reflects on how the inscription of mental health nurses and their managers within the "accountability culture" might affect the status and significance of clinical supervision in mental health nursing. It also discusses how the broader organizational and cultural context may shape clinical supervision | Tendency of supervisees to manage the supervision agenda by focusing on ‘safe talk' means that they simultaneously related to direct client care; talk’ means that they simultaneously avoid dialogues; mistrust; given low priority, not seen as 'real work'; used to control & discipline |  | Commentary |
| Gray | 2001 | UK | N/A | N/A | Analyses relationships between clinical and management supervision. | Historical development suggests that management and clinical supervision are more functionally connected than many would believe, even though UKCC asserts that clinical supervision is not a managerial control system. With this in mind, the blurred boundaries between line management and clinical supervision are seen as problematic. as is confidentiality, venues for supervision, cost |  | Discussion Paper |
| Hadfield | 2000 | England & Scotland | Children's Services | 12 nurses | Gain an understanding from the ’users’ perspective of the impact of clinical supervision on paediatric nurses’ practice | Main barrier as time commitment for clinical supervision as it is a labour-intensive activity. Supervisees reported experiencing anxiety about taking time for clinical supervision and having difficulty finding time |  | Research (Qualitative) |
| Howard and Eddy-Imishue | 2020 | England | Mental Health | N/A | To explore factors which influence adequate and effective clinical supervision for inpatient mental health nurses' personal and professional development. | Negative previous experience of clinical supervision; clinical supervision not perceived as 'proper work'; lack of priority given to clinical supervision; lack of knowledge about implementing & sustaining clinical supervision; clinical supervision induced stress; concerns about uncovering mismanagement through clinical supervision session; anticipated lack of support from managers; parallel forums as alternatives to clinical supervision; time constraints and staffing levels; conflation of clinical supervision and line management; too much being asked of clinical supervision as a process; negatively labelled when open & transparent about vulnerabilities; power dynamic clinical supervision; impractical within environment routine; not enough choice of supervisor; workload | Making staff feel valued & supported enabled better coping therefore less stress; facilitated job satisfaction; opportunity to learn about personal values; clinical supervision facilitating new perspectives on problems; reducing emotional burden; opportunity for reflection & in-depth exploration; when defined by the nurses who participate in it; organisational ownership; pre-supervision course/intervention | Literature Review |
| Howatson-Jones | 2003 | N/A | N/A | N/A | To explore context of clinical supervision and difficulties in implementing clinical supervision |  | Working more creatively using technology. Using existing forums. Networking with other disciplines. | Discussion Paper |
| Hughes & Morcom | 1998 | Not specified | Mental Health Inpatient | 14 | To determine those factors which influenced the effectiveness (or not) of the clinical supervision system within the mental health in-patient area of the trust. | Lack of interest and understanding, active ridicule, and working with colleagues who were suspicious and dismissive. Staff shortages, not being able to leave the ward when in charge, and the unpredictable nature of the work. Changed shifts and shift patterns, non-availability of rooms, staff sickness | Positive and encouraging attitude to clinical supervision by the clinical manager (which helped alleviate some of the practical difficulties), high degree of motivation of both supervisors and supervisees. Sympathetic managers to ensure staff cover was arranged, and shift patterns could be altered to enable staff to participate in clinical supervision. Ensuring personal time was repaid to staff. | Research (Longitudinal Study) |
| Johansson et al., | 2006 | Norway | N/A | 13 | To explore & describe nurse supervisors' view of caring in nursing supervision& how to make it visible |  | Consciousness of value base, having a relationship characterised by learning & consolation, having a caring supervisor | Research |
| Jones-Berry | 2018 | England | N/A | N/A | To explore nurses’ wellbeing & clinical supervision | Time & lack of staffing |  | Commentary |
| Keegan | 2013 | New Zealand | Various- practice, hospice & dementia care nurses | N/A | To explore how to make supervision work | Inherent trust issues | location (quiet, comfortable nonclinical), scheduled as work, mutual respect, supportive management, supervisor expertise, agreed ground rules, fixed weekly sessions where attendance is compulsory to attend, | Discussion paper |
| Kelly et al., | 2001 | Northern Ireland | Mental health | 153 | To provide insight into clinical supervision experiences | Education & training deficits; interface between managerial & clinical supervision; lack of clarity about underpinning concepts of clinical supervision |  | Research (Survey) |
| Koivu et al., | 2011a | Finland | Medical- Surgical Registered & Assistant Nurses | 328 | To identify which nurses, decide to participate in clinical supervision when it is provided for all nursing staff | Diminished professional efficacy and cynical attitudes towards work hindered participation in clinical supervision | Qualities of self-confidence, commitment and competence | Research (Survey) |
| Koivu et al., | 2011b | Finland | Medical- Surgical Registered & Assistant Nurses | 328 | To provide a more nuanced comparison of uptake of clinical supervision (in medical vs surgical unit staff) | On the medical units, the uptake of clinical supervision was linked to worse mastery at work and more symptoms of distress and burnout. difficulty establishing common ground and struggles between nurses may have influenced how clinical supervision was undertaken | Involvement in clinical supervision on the surgical units was strongly associated with the perceptions of better mastery at work, more positive assessments of ward culture and fewer symptoms of burnout. | Research (Survey) |
| Lister & Crisp | 2005 | Scotland | Community Nurses (health visitors, district nurses, sexual health nurses, learning disability & community mental health nurses, public health practitioners, substance use) | 99 | To explore community nurses’ and health care managers’ understanding and experience of clinical supervision in child protection | Nurses confused case discussions with clinical supervision. Lack of clarity on purpose and nature of clinical supervision. Nurses 'avoided clinical supervision like the plague' because they associated it with performance appraisal or caseload management and was about doing wrong, culture of 'getting on with it' and not revealing problems, equating asking for help with not coping | Legitimising clinical supervision as a right rather than an imposition | Research (Interviews) |
| Long et al., | 2014 | Unclear | Medium secure mental health nursing setting | 128 | To investigate perceived benefits of clinical supervision; best practice elements of clinical supervision; practical aspects of clinical supervision and how to improve the practice of clinical supervision | Fear of victimization, thinking their supervisor will not listen, feeling embarrassed about needs, personal problems, fear of issues reflecting badly on them, peer pressure, not wanting to cause conflict and not practising within the job role. |  | Research (Questionnaires) |
| Lowry | 1998 | N/A | N/A | N/A | To examine roles and responsibilities in clinical supervision and discuss the benefits for individuals and for health services. | The misapprehension that clinical supervision is fault finding, management driven or a tool for enhancing professional status are considered barriers |  | Discussion Paper |
| Lyon | 1998 | Unclear | Acute setting | Unlcear | Using Hart and Bond’s action research typology to clarify implementation of clinical supervision; the aims were to Generate an appropriate process of clinical supervision; Identify contextual factors which contributed to or inhibited the implementation of clinical supervision; Identify structures necessary for the ongoing support of clinical supervision |  | Having structures that support implementation | Research (Action Research) |
| Malin | 2000 | England | Learning Disability Community Homes & Teams | 7 registered nurses and 4 community team members (including psychology, social worker) | To examine how clinical supervision was operating, its strengths, its weaknesses and where improvements might be made | Nurses expressing apprehension or unpreparedness. A perceived general concern over the relatively low status of clinical supervision (thought to be due to absence of visible management approval or failure to articulate properly the objective of supervision). The `paperwork' involved presented as an ambiguous threat to some nursing staff who believed that what was written down could in fact be used against them | Having policies and practices explained to nurses and having feelings of reassurance served as a facilitator to accessing clinical supervision | Research (Qualitative) |
| McCarron et al., | 2017 | UK | Secure adolescent service | 49 Healthcare Assistants and 20 Nurses | To explore experience of, and access to, clinical supervision for nurses and healthcare assistants. To outline barriers to obtaining clinical supervision. To outline the perceptions of staff around the value of clinical supervision. To identify whether healthcare assistants can reflect on the purpose of clinical supervision and identify consequences of inadequate clinical supervision |  | Intervention which included- Raising staff awareness of supervision. 2. An introduction of multidisciplinary supervision (as suggested by Mullarkey, Keeley, & Playle, 2001). 3. An increase in group supervision. 4. Increased emphasis was placed on individuals acting as “pushing forces” (Driscoll, 2000) to promote clinical supervision. 5. Increased monitoring of supervision rates (as suggested by Rafferty, Llewellyn-Davies, & Hewitt, 2000) at a board level via a quality dashboard. 6. A push via all line managers to ensure that supervision happened and was documented via a quality compliance manager (QCM) system. 7. Absences in supervision were chased up quickly via the QCM implemented with good effect on clinical supervision experience for staff access to and culture around clinical supervision also improved. | Research (Qualitative) |
| O'connell et al., | 2011 | Australia | General Nurses acute wards maternity, medical & gastroenterology ward | 36 staff (enrolled nurses, registered nurses & midwives) pre; 27 posts, 20 focus group. | Outline the practicalities of implementing team clinical supervision in acute wards maternity, medical & gastroenterology ward | Finding time amidst unpredictable and hectic nature of work, attending clinical supervision hindered completion of other routine tasks |  | Research (Mixed Methods) |
| Puffett & Perkins | 2017 | England | Palliative Care Nurses | 2 focus groups comprising 10 participants (6 RN, 2HCA’s engaged in clinical supervision) + (1RN & 1HCA not engaged in clinical supervision) | Investigate what influences palliative care nurses in their choice to engage in or decline clinical supervision. | Fear of clinical supervision use for when something goes wrong, confused with “appraisal”, confusing term and mystic of clinical supervision, lack of understanding and clarity about what it is. | Having choice of either group or individual clinical supervision helps engagement | Research (Focus Groups) |
| Rice et al., | 2007 | Northern Ireland | Mental Health Nursing | Unclear | Explore ways to make clinical supervision available to all mental health nurses and in doing so to evaluate and improve their contribution to patient care | Less enthusiasm among hard pressed clinical staff who were facing regular alterations in staff numbers and expertise. Absence of regional guidelines on its development and introduction; apprehension and fear of engaging in clinical supervision among mental health nurses. Definition of clinical supervision problematic, confusion about what clinical supervision is and how it relates to other supervisory approaches | Use of a combination of individual and group supervision. Need for adequate financial resources. Time - each practitioner needs between 1.5 and 2 hours protected time per month. | Research (Survey) |
| Robinson | 2005 | Not specified | Children’s nursing- Acute paediatric ward | Not specified | Describe implementation of clinical supervision in an acute paediatric ward by introducing the role of a clinical supervision facilitator. | Lack of time affects ability to access regular clinical supervision. Supervisors identified but not available | Need to choose supervisor/supervisee partnerships and establish supervisory relationships. Training | Research (Implementation Study |
| Sexton-Bradshaw | 1999 | England | Children’s nursing NICU & PICU | 10 NICU staff (with experience of 1:1 clinical supervision); 15 PICU staff (without experience of clinical supervision. 6 NICU nurses interviewed regarding process understanding. | To explore the experiences and nurses’ perception of the value of clinical supervision | Confusion over terms | Choice of supervisor, support (access and time), training | Research (Qualitative) |
| Sines & McNally | 2007 | England | Learning Disability Nursing | 35 | To explore the extent to which registered learning disability nurses working in a range of residential care settings in an area of south-east England engaged in clinical supervision and to investigate their perceptions and experiences of these encounters |  | Greater clarity in the roles of supervisor and supervisee, and a clearer separation of managerial and developmental imperatives. Support and preparation for supervision, and range of options for type of supervision and choice of supervisor. Setting aside dedicated time for supervision. | Research (Questionnaires) |
| Sloan | 1999b | Unclear | Mental Health Nursing | 8 | To provide a community mental health nurse perspective on the good characteristics of a clinical supervisor | Having clinical supervisor allocated and the supervisor also being their manager, having supervision sessions documented and stored by the manager threatened the full utility of the clinical supervision |  | Research (Mixed Methods) |
| Smith | 2001 | England | General Nursing | 11 | To explore why clinical supervision is so difficult to introduce and sustain in general nursing | NHS culture, lack of time, the belief that clinical supervision was already done on informal basis was a barrier to doing this formally, it was viewed as another form of appraisal and another management tool, hidden agendas, negative attitudes | Having choice of clinical supervisor | Research |
| Spence et al., | 2002 | UK | Diverse fields- including mental health, general nurses, learning disability, elderly care specialist nurses | 188 (pre-test survey) 154 (post-test survey), plus 4 focus groups (with 27 participants between them) | To describe the project development and implementation of a collaborative approach to implementing clinical supervision between PCT & school of nursing and midwifery (including educationalists, clinicians and managers from lead steering group) | Time | For this study, the group supervision format was a facilitator of clinical supervision because it suited the team needs. | Research (Mixed Methods) |
| Stevenson & Jackson | 2000 | England | CPN's | 8 | To produce local knowledge of clinical supervision, with a desire to explore a different style of clinical supervision called Egalitarian Consultation Meetings. Aim of the study was to provide a description and particular theory of the social construction of clinical supervision set in a postmodern context- re-constructing clinical supervision as Egalitarian Consultation Meetings | Radical equated to dangerous in terms of the watchful organization and a return to ‘real’ work (case supervision) was observed. | Innovation in relation to clinical supervision may benefit from a change in institutional culture | Research |
| Turner et al., | 2005 | N/A | Neurology- general nursing | 5 | To outline the salient features of how effective group supervision can be achieved, what are the main pitfalls/ barriers and how to overcome them |  | Having a supervisor who doesn’t need to be nurse but should be an experienced clinician with knowledge of healthcare organisations and with supervisory expertise | Discussion Paper |
| White & Winstanley | 2010 | Australia | Mental Health Nurses | 17 | To explore substantive issues related to the implementation of Clinical Supervision (clinical supervision) (from RCT in 2009a+b) | Suspiciousness, "tautological maelstrom”, confusion generated by lack of clear operational definition & conceptual ignorance. Less staff commitment because of reservations about the purpose of clinical supervision. Dispassionate resignation to the actualities of the existing health care system | Because of the belief that clinical supervision reduced feelings of powerlessness and frustration, staff also felt that clinical supervision reduced the risk of burning out and long-term illness | Research (Qualitative) |
| Williams & Irvine | 2009 | UK | Nursing- field not specified | 12 | To explore the nature of the nurse clinical supervisor role | Lack of guidelines fulfilling the role, gaps in the structure of the clinical supervisor’s role, complexities in the training and support of nurse clinical supervisors. Time. Inconsistencies within the process of clinical supervision which could inhibit nursing staff from engaging in supervision. |  | Research (Qualitative) |

**Table 3: Literature Review Question 2- Organisational Barriers and Facilitators to Implementing Clinical Supervision**

| Author/s | Year Published | Country / Region | Nursing Discipline/Setting | Number of Participants | Aim/s | Barriers | Facilitators | Type of Paper |
| --- | --- | --- | --- | --- | --- | --- | --- | --- |
| Ainsworth | 2000 | UK | Orthopaedics clinical  supervision | N/A | Meeting the challenges of clinical supervision |  | Nurses valuing themselves, recognition of the need for time to reflect on the realities of care delivery; Facilitation by knowledgeable practitioner irrespective of grade to move away from traditional hierarchy towards a partnership model; Commitment and clinical leadership to support clinical supervision, clinical supervision prerequisite for nurses, clinical supervision condition for commissioning/contracting care, measure of clinical effectiveness. | Opinion Piece |
| Brunero | 2012 | Australia | Nurse managers, nurses, general nurses | 9 groups | To review the implementation of clinical supervision across several different nursing specialities | Time away from clinical demands, physical space, ongoing training costs, availability of facilitators | Nurse managers taking on a clinical role to allow nurses to access clinical supervision, nurses being involved in developmental process of clinical supervision implementation strategy so that they could take ownership of it, handbook explaining roles, functions, purpose of clinical supervision, developing champions of clinical supervision within the workplace | Evaluation |
| Bryant | 2010 | N/A | N/A | N/A | Provides an introduction to clinical supervision |  | Contract; protected time; supervisor skills; 'buy-in' from participants | Discussion Paper |
| Bush | 2005 | N/A | N/A | N/A | Discusses the barriers and the reasons for them and overcoming them. | Managers concerned about loss of power, control & authority if someone other than them conducts clinical supervision; Barrier's stem from political conflict, lack of understanding, constraints of time and resources including appropriate environment. Nursing itself is resistant to change. |  | Discussion Paper |
| Butterworth et al., | 2008 | UK | N/A | N/A | Analyse themes from literature (2001-2007); describe emerging trends& outcomes | Organisational culture | Technological advancement -Telephone clinical supervision scheme, video conferencing; organisational culture | Literature Review |
| Buus et al., | 2018 | Denmark | Mental Health Nursing | 24 | To examine resistance to group clinical supervision by interviewing nurses who did not participate in clinical supervision | Managers not fully committed, no consequence for non-participation |  | Research (Qualitative) |
| Buus et al., | 2013 | Danish | Psychiatric Nursing- wards | Ward A- 5, 4, & 3 participants @ three test sessions. Ward B- 4, 3, & 4 | Explain the development, implementation, and adjustment of the manual for the intervention, describe the content and structure of the final educational intervention, and reflect on six key-issues in relation to implementing the manual. | Where the clinical supervision relationship was an authoritative one, participants were hesitant in voicing their own thoughts and opinions | Sensitivity of the group supervisor to potential issues of shame and fear of self-disclosure among participants is an important facilitator. | Research (Implementation Study) |
| Buus et al., | 2011 | Danish study | Psychiatric Nurse reflection on clinical supervision | 22 | To explore psychiatric hospital nursing staff reflections on participating in clinical supervision | High workloads, lack of continuity compromising eventual effect, limited clinical value and impact, passing benefits - not sustained, expectation to attend on day off - not built into daily work. |  | Research (Interviews) |
| Cairns | 1998 | N/A | N/A | N/A | Discusses how practice nurses can make a considerable contribution to the development and delivery of primary care services. | Term 'supervisor' is traditionally associated with industry and commerce and is linked conceptually to a controlling authority, this may be a barrier to its effective implementation. practice development is dominated by medical, nursing and managerial hierarchies! With so many hierarchies already struggling to control and direct can the simple process of reflection with a peer ever carry enough power to influence practice. | remove practitioner suspicion and develop useable local models otherwise it won’t be used by those who need this most. Mix disciplines to create more capacity for clinical supervision | Discussion Paper |
| Cheater & Hale | 2001 | England | Practice Nurses | Unclear - (states all practice nurses in Leicestershire) | Evaluation of scheme - Assess the level of uptake of clinical supervision by practice nurses; identify factors which hindered/facilitated uptake; evaluate how far clinical supervision had influenced i) quality of clinical care ii) organisation of care iii) professional development. | Lack of protected time, misconceptions clinical supervision linked to poor performance, |  | Evaluation |
| Chilvers and Ramsey | 2009 | England | Hospice | Not specified | Practicalities of setting up clinical supervision for RNs,HCAs & community associate practitioners (CAPs) across 3 hospice sites. | Insufficient numbers of supervisors, lack of time, clinical area too busy |  | Literature Review |
| Cleary & Freeman | 2006 | N/A | mental health nursing context | n/a | To outline some pragmatic issues concerning clinical supervision and advocate for facility-specific professional development and support processes that incorporate succession planning opportunities | Organisational culture a key limitation to the widespread implementation of clinical supervision. o Few organisations are prepared to pay for supervisors or to recruit additional staff for the sole purpose of clinical supervision. Work rostering can make it difficult to prioritise time for clinical supervision. o Places free of distraction and interruption are next to impossible to find, most facilities do not have available rooms. Supervisors overwhelmed by other priorities. | Clinical supervision is contingent on additional resources, managerial support, nurse motivation, and the opportunity to leave and be covered in the workplace. | Discussion paper |
| Colthart et al., | 2018 | N/A | N/A | N/A | Providing practical steps for implementing clinical supervision. Useful in terms of current context and some current drivers for implementing supervision. |  | Organisational endorsement and nurse managers engagement key to supporting effective clinical supervision. organisations should have a policy to support clinical supervision, including commitment, purpose and framework for supervision. A model for supervision across the whole organisation also recommended, but no specific recommendation as to model. | Discussion Paper |
| Cotton | 2001 | N/A | N/A | N/A | Utilising a Foucauldian approach to discourse, power-knowledge and clinical gaze, the paper radically interrogates this seemingly unproblematic conceptualisation of clinical supervision and its rising popularity and influence | UK style of clinical supervision appears ambiguous and confusing, even to those seeking to implement it in its place of origin. promotion appears to be largely based on rhetoric rather than adequate evidence of benefits to clinical nurses and nursing. the discourse of clinical supervision marginalises clinical nurses and patients and subjugates their knowledges. |  | Discussion Paper |
| Cross et al., | 2010 | Australia | General Nursing (acute medical ward) | 6 ANUMs participated in 15 group sessions in total and 4 took part in the focus group. | To implement and evaluate group clinical supervision for associate nurse unit managers (ANUMs) in a busy medical ward. | Questions whether word ‘supervision’ acceptable, or more problematic as signifying ‘oversight’ and ‘watching’. | Need for dedicated time | Evaluation (Pilot Study) |
| Darley | 2001 | N/A | Medium-secure psychiatric unit | N/A | Description of the practice of clinical supervision in author’s own practice area | Clinical supervision presented as having a mystical power that 'supervisors' take on and then bestow upon those who are 'supervisees' | There is a need to exercise clinical supervision of this mystical power and its role and purpose must be restated. Likens clinical supervision to good management, and therefore, the integration of supervision into everyday working practice using the principles of good management is desirable. Discusses benefits of using a 'four aspects model' for supervision | Discussion Paper |
| Fowler | 2013c | N/A | N/A | N/A | Clinical supervision: Implementation at a strategic level | No strategic planning: No budget attached to development & implementation; No strategic leadership or ownership; Outcomes not defined; Process too ambitious; No built-in monitoring or evaluation; Professional & clinical groups fail to agree definition; Clinical staff & middle managers not involved in the development therefore again no ownership; Process not integrated into strategic communication systems; Managerial ground rules not agreed e.g., time commitments. |  | Discussion Paper (part of a series) |
| Freshwater et al., | 2003 | England | Prisons | 35 | Establish and evaluate a strategy for the effective implementation of clinical supervision within a group of prisons and subsequently to make recommendations for good practice, identifying real and perceived barriers. | Cultural and institutional issues such as suspicion and cynicism as barriers to implementation. Organisational difficulties, for example, shift patterns and low staffing levels |  | Research (Mixed Methods) |
| Gonge & Buus | 2016 | Danish study | Psychiatric nursing | 115 psychiatric nursing staff | (a) To test whether a meta- practice intervention could increase psychiatric nurses’ clinical supervision participation and effectiveness of existing supervision practices, and (b) To explore organizational constraints to implementation of these strengthened practices. | The lower participation and effectiveness of clinical supervision was due to largescale organisation change which impacted the intervention group. diminishing experience of social support from colleagues was associated with reduced participation in clinical supervision, while b) additional quantitative demands were associated with staff reporting difficulties finding time for supervision. | Implementation of clinical supervision must at the outset enjoy full organizational support as organizational constraints are otherwise likely to obstruct the implementation | Research (Longitudinal Study) |
| Gonge & Buus | 2010 | Danish study | Psychiatric Nursing | 239 | To investigate how often psychiatric nursing staff participate in clinical supervision and any possible associations among individual and workplace factors that influence participation | Characteristic clinical supervision of the workplace, including organisational location, work shift, and work-environmental factors, are related to participation and may affect the outcome of clinical supervision. |  | Research (Survey) |
| Grant & Townend | 2007 | UK | Mental Health Nursing | N/A | Paper reflects on how the inscription of mental health nurses and their managers within the "accountability culture" might affect the status and significance of clinical supervision in mental health nursing. It also discusses how the broader organizational and cultural context may shape clinical supervision. | In trust aversive organizational cultures, the reflective practice function of clinical supervision may be viewed as not constituting real work by managers and therefore given a low priority | Having a less risk aversive culture, and a more learning-based culture. Leadership to ensure the right structures are in place to support clinical supervision | Commentary |
| Gray | 2001 | UK | N/A | N/A | Analyses relationships between clinical and management supervision. | Historical development suggests that management and clinical supervision are more functionally connected than many would believe, even though UKCC asserts that clinical supervision is not a managerial control system. With this in mind, the blurred boundaries between line management and clinical supervision are seen as problematic. as is confidentiality, venues for supervision, cost |  | Discussion Paper |
| Hadfield | 2000 | England & Scotland | Children's Services | 12 nurses | Gain an understanding from the ’users’ perspective of the impact of clinical supervision on paediatric nurses’ practice. | main barrier as time commitment for clinical supervision as it is a labour-intensive activity | There was a recommendation for formal linkage to PREP (Post Registration Education and Practice) as a means of getting the status, time and resources needed for clinical supervision. | Research (Qualitative) |
| Hancox et al., | 2004 | Australia | Mental Health nursing context | 63 | Evaluation of educational programme on clinical supervision | Inadequate training |  | Evaluation |
| Howatson-Jones | 2003 | N/A | N/A | N/A | Exploring context of clinical supervision and difficulties in implementing clinical supervision. |  | Working more creatively using technology. Using existing forums. Networking with other disciplines. | Discussion Paper |
| Jenkins et al., | 2000 | West Wales | Various | 35 | Audit clinical supervision experience | Time, lack of organisational support, environment, supervision relationships. | Knowledge, learning, support, recognition of challenges, mutual contracts. | Research |
| Keegan | 2013 | New Zealand | Various- practice, hospice & dementia care nurses | N/A | Exploring how to make supervision work | Space, time, money, & skill base of supervisors, clinical supervision as organizational surveillance, organizational pressure on nurses to make time for clinical supervision, clinical supervision being ill-defined. |  | Discussion paper |
| Koivu et al., | 2011a | Finland | Medical- Surgical Registered & Assistant Nurses | 328 | Identify which nurses decide to participate in clinical supervision (clinical supervision) when it is provided for all nursing staff |  | Supportive and proactive leadership hallmark of a more successfully rolled out clinical supervision. Empowering and fair leadership | Research (Survey) |
| Koivu et al., | 2011b | Finland | Medical- Surgical Registered & Assistant Nurses | 328 | More nuanced comparison of uptake of clinical supervision (medical vs surgical unit staff) |  | Involvement in clinical supervision on the surgical units was strongly associated with more positive assessments of ward culture and fewer symptoms of burnout. nurse managers on the surgical units had succeeded at empowering healthy, self-confident and innovative nurses while arranging clinical supervision for staff. | Research (Survey) |
| Lister & Crisp | 2005 | Scotland | Community Nurses (health visitors, district nurses, family planning & sexual health nurses, learning disability nurses & community mental health nurses, public health practitioners, substance use) | 99 | To explore community nurses’ and health care managers’ understanding and experience of clinical supervision in child protection | Concerns that clinical supervision is somehow antithetical to professional autonomy. Health service culture of ‘an unholy alliance of cost-cutting and professional autonomy’. the historical difficulties regarding supervision were attributed to several aspects of nursing culture. |  | Research (Interviews) |
| Long et al., | 2014 | not explicitly stated | medium secure mental health nursing | 128 | To investigate 1. Perceived benefits of clinical supervision 2. Best practice elements of clinical supervision 3. Practical aspects of clinical supervision including meeting standards, meeting learning needs, issues in supervision and how to improve the practice of clinical supervision | Fear of victimization, thinking their supervisor will not listen. Lack of trust, feeling embarrassed about needs, personal problems, fear of issues reflecting badly on them, peer pressure, not wanting to cause conflict and not practising within the job role. time for both supervisor and supervisee, work pressures, finding a suitable supervisor, attitude of the supervisor, lack of trust, unapproachable supervisor, feeling uncomfortable with supervisor and confidentiality issues. consistency, teamwork and training, lack of contact with supervisor. | Having more regular clinical supervision, a list of available supervisors, protected time to meet the supervisor, ward to be staffed better to allow for clinical supervision a supervisor from the same profession | Research (Questionnaires) |
| Lowry | 1998 | N/A | N/A | N/A | Examines roles and responsibilities in clinical supervision and discusses the benefits for individuals and for health services. | Resource implications of any changes to nursing practice, especially those that take the nurse away from the patient | A national standard for clinical supervision, perhaps built into the PREP framework, could better serve the needs of individual practitioners. | Discussion Paper |
| Lynch & Happell | 2008 | Australia | rural mental health service (nurses) | Documentation audit plus 7 interviews with nurses involved in clinical supervision implementation | Exploring the actual implementation of clinical supervision in one rural mental health se |  | Structure plus good leader to hold everything together. development and enactment of a strategic plan. Addressing the culture of the organization and education and training. Education to dispel the myths commonly associated with clinical supervision and education needs to be ongoing | Research (Interviews and Documentation Audit) |
| Malin | 2000 | England | Learning Disability Community Homes & Teams | 7 registered nurses and 4 community team members (including psychology, social worker) | Aim was to examine how clinical supervision was operating, its strengths, its weaknesses and where improvements might be made | Low status of clinical supervision, thought to be due to absence of visible management approval or failure to articulate properly the objective of supervision. management failure to endorse supervision more fully and to create an infrastructure for it to happen. | Offering staff education | Research (Qualitative) |
| McCarron et al., | 2017 | UK | secure adolescent service | 49 HCA's, 20 Nurses | Experience of, and access to, clinical supervision for nurses and HCAs. 2. Barriers to obtaining clinical supervision. 3. The perceptions of staff around the value of clinical supervision. 4. Whether HCAs can reflect on the purpose of clinical supervision, and identify consequences of inadequate clinical supervision | Time and staffing issues were the most commonly perceived barriers to adequate supervision | Intervention which included - 1. Raising staff awareness of supervision. 2. An introduction of multidisciplinary supervision (as suggested by Mullarkey, Keeley, & Playle, 2001). 3. An increase in group supervision. 4. Increased emphasis was placed on individuals acting as “pushing forces” (Driscoll, 2000) to promote clinical supervision. 5. Increased monitoring of supervision rates (as suggested by Rafferty, Llewellyn-Davies, & Hewitt, 2000) at a board level via a quality dashboard. 6. A push via all line managers to ensure that supervision happened and was documented via a quality compliance manager (QCM) system. 7. Absences in supervision were chased up quickly via the QCM implemented with good effect on clinical supervision experience for staff. access to and culture around clinical supervision also improved. | Research (Mixed Methods) |
| O'connell et al., | 2011 | Australia | General Nurses acute wards maternity, medical & gastroenterology ward | 36 staff (enrolled nurses, registered nurses & midwives) pre, 27 post, 20 focus grp. | Practicalities of implementing team clinical supervision in acute wards maternity, medical & gastroenterology ward |  | Clinical supervision ambassador to promote, rotate days for diff staff to attend | Research (Mixed Methods) |
| Olsson et al., | 1998 | not explicitly stated | Homecare staff caring for people with dementia- included home helpers, home care manager, social worker, mental health nurses, district nurses, assistant nurses, registered nurses, those with no vocational training | 63, (11 interviews and 52 questionnaires) | Investigate views on structure, content and effects of systematic clinical supervision. Homecare staff in dementia care | Reorganizations, staff turnover, new supervisors, the varying number of supervisory sessions and whether the staff was involved in the care of dementia clients | Systematic Clinical Supervision requires stable structure, continuity and restricted admission to new members; a work climate where feelings as well as practical problems are dealt with; that the participants present their own cases, are well-prepared and have practical experience of the type of care focused by the clinical supervision; a specialist organization for staff with no training in the field concerned | Research (Mixed Methods) |
| Puffett & Perkins | 2017 | England | Palliative Care Nurses | 2 focus groups comprising 10 participants (6 RN, 2HCA’s engaged in clinical supervision) + (1RN & 1HCA not engaged in clinical supervision) | To investigate what influences palliative care nurses in their choice to engage in or decline clinical supervision. |  | Protected time was essential for staff to be able to engage in clinical supervision. Staff who worked in larger teams reported higher levels of engagement, whereas a small team reported less need due to more informal team support | Research (Focus Groups) |
| Rice et al., | 2007 | Northern Ireland | Mental Health Nursing | Unclear | Explore ways to make clinical supervision available to all mental health nurses and in doing so to evaluate and improve their contribution to patient care | Less enthusiasm among hard pressed clinical staff who were facing regular alterations in staff numbers and expertise. absence of regional guidelines on its development and introduction | The preferred model was a combination of individual and group supervision. adequate financial resource. Time - each practitioner needs between 1.5 and 2 h protected time per month. | Research (Survey) |
| Robinson | 2005 | Not explicitly stated | Children’s nursing- Acute paediatric ward | Not specified | Description of implementation of clinical supervision in an acute paediatric ward by introducing the role of a clinical supervision facilitator. | Lack of time affects ability to access regular clinical supervision. Supervisors identified but not available | Need to choose supervisor/supervisee partnerships and establish supervisory relationships. Need for training | Research (Implementation Study) |
| Severinsson & Hallberg | 1996 | Sweden | Registered nurses (psychiatric care, medical care, surgery, primary health, midwifery specialisms) | 18 | Investigate nurse supervisors' views of their supervisory styles, their personal qualities and their leadership role, and the development of these styles |  | Leadership with high values on technique of clinical supervision, techniques in providing clinical supervision, responsibility for facilitating process, responsibility for creating a climate conducive to supervision and focusing on the main themes. | Research (Mixed Methods) |
| Spence et al., | 2002 | UK | Diverse fields- including mental health, general nurses, learning disability, elderly care specialist nurses | 188 (pre-test survey) 154 (post-test survey), plus 4 focus groups (with 27 participants between them) | Paper describes the project development and implementation of a collaborative approach to implementing clinical supervision between PCT & school of nursing and midwifery (including educationalists, clinicians and managers from lead steering group) | Administrative support. Culture of the areas and responsiveness of the groups to change | Collaborative approach (between pct and school of nursing & midwifery). The project design pre-empted what would have been potential barriers (as identified in existing literature at the time) so it ensured rewards (personal professional development, confidence), training in collaborative skills done, shared resources and partnership with the community across organizational and professional boundaries, shared commitment and shared credit for the project by all who participated and neutral base of operation. | Research (Mixed Methods) |
| Stacey et al., | 2020 | UK | Cross discipline | 266 | To explore the acceptability, feasibility, and experience of Resilience Based Clinical Supervision to support transition to practice in newly qualified nurses. | Requires a mental health qualification in order to have the skills to respond to and contain the distress or emotional disclosure shared especially in large groups with inconsistent membership. questioned the value of a focus on emotions also. expressed nervousness about the potential for the model to encourage a paternalist response. | Clear structure for facilitators to receive their own supervision, Organisational commitment, Clear processes for escalation of workplace issues, Clear processes for escalation of safeguarding issues relating to staff wellbeing, Widespread implementation | Research (Qualitative) |
| Stevenson | 2005 | England | Nursing | N/A | Paper sought to challenge existing ideas about clinical supervision and to construct an alternative approach, enact a different kind of clinical supervision, known as Egalitarian Consultation Meetings (ECM) |  | Concludes that no approach is without a political dimension and the ECM group was not immune to the broader, modernist culture. Thus, change in clinical supervision practices needs to be at multiple levels of the system | Discussion Paper |
| Temane et al., | 2014 | South Africa | Advanced Psychiatric Nursing | 8 | Explores and describes advanced psychiatric nurse practitioners’ ideas and needs regarding clinical supervision in private practice |  | A culture of support needs to be fostered in mental health settings for psychiatric nurse practitioners and advanced psychiatric nurse practitioners to enhance professional practice | Research (Qualitative) |
| Tobias et al., | 2016 | England | Community and inpatient settings in a mental health and learning disability NHS Trust | 176 (online survey) then 8 focus groups | To improve understanding of supervision provision at an adult mental health and learning disability directorate and use findings to overcome barriers to clinical supervision | Nurses value clinical supervision but time, interruptions and lack of a suitable  environment, as well as lack of  structured support are barriers to accessing it. Confusion around confidentiality of clinical supervision records which contributes to reluctance to record supervision details on the electronic system. | Clear standards for supervision. Ensuring staff have clarity about who has access to their documentation/clinical supervision records | Research (Mixed Methods) |
| Webb | 1997 | England | Community Healthcare Trust covers adult mental health services, elderly mental health services, learning disability, community nurses, palliative care, health visiting and physical disability, | 1 community healthcare trust | Evaluates a training course designed to help with the implementation of an NHS Trust's clinical supervision policy | Clinical supervision more difficult to implement in some settings (like health visiting and district nursing) more than others- e.g., because of large geographical areas. Sessions used for management rather than clinical issues. No identified time for clinical supervision. Imposed managerial clinical supervision. Difficulty finding a clinical supervisor. lack of individuals who have completed the clinical supervision training to be able to implement it. |  | Evaluation /Audit |
| White & Winstanley | 2006 | New Zealand & Australia | Registered Nurses, Clinical Nurse Specialists, Nurse Consultants, Nurse Unit Managers | 146 | Financial modelling study exploring the resource and management issues in introducing and maintaining a clinical supervision programme for nurses |  | Financial modelling suggests on average, the cost of giving peer group one-to-one supervision to a RN represented a cost of about 1% of the nurse’s annual salary- and given the costs of burnout etc, this level of investment was justified given clinical supervision effects on reducing burnout | Financial Modelling |
| White & Winstanley | 2009b | Australia | Mental Health Nurses | 139 diary accounts | Qualitative account of issues involved in the implementation of nursing Clinical Supervision (clinical supervision) in mental health settings, sits within the wider context of a novel Australian randomized controlled trial (RCT) in White & Winstanley 2009a) | Interruptions during clinical supervision sessions, lateness and other competing demands on the supervisor’s and supervisee’s clinical time and other in-service training activity start-up difficulties were not alleviated in settings where the prevailing management culture was unsupportive, obstructive or, on occasion, frankly hostile to the local clinical supervision innovation. Query longevity beyond the trial period | Managerial support, confidentiality | Research (Diary Accounts) |
| White & Winstanley | 2010 | Australia | Mental Health Nurses | 17 | Explore substantive issues related to the implementation of Clinical Supervision (clinical supervision) (from RCT in 2009a+b) | Cost burden if taken as additional activity, org culture. Senior managers embraced clinical supervision and were disappointed when junior managerial colleagues did not hold a similar conviction, when tested by the realities of clinical supervision implementation. If clinical supervision was regarded as an additional activity, it stretched human resources and created inter-staff tensions. | A single, cohesive and explicit management position on clinical supervision in each Health Service entity may obviate some of the impediments to clinical supervision implementation. | Research (Qualitative) |
| White & Winstanley | 2009a | Australia | Mental Health Nurses | Intervention Arm- 240; Control Arm- 170 | Establish the effects of providing regular group clinical supervision to community and inpatient-based nursing staff, working in mental health settings in Queensland | If clinical supervision is poorly understood at the conceptual level and delivered superficially in practice- at best, it may waste public money and other scarce resources or, at worst, prove ineffectual and/or inadvertently detrimental to supervise and health service consumer alike. If the clinical supervision policy implementation is located within a culture which is antithetical to the endeavour, it will fail. Time- as work gets busier, those who need clinical supervision most don’t get it | Develop a mind-set at all levels of the workforce that comprehends clinical supervision as bona fide work innovation, not an activity which is different from the work | Research (Trial Design) |
| Williams & Irvine | 2009 | UK | Not specified | 12 | Explore the nature of the nurse clinical supervisor role |  | Managerial support in the form of prioritizing training and offering support mechanisms help nurses to effectively fulfil the clinical supervisor role | Research (Qualitative) |
| Williams et al., | 2005 | England | Community Nurses from 3 primary care trusts | 45 | Describes how community nurses interpreted and adapted a formal system of clinical supervision to suit their specific needs | Challenge presented by trying to protect time- particularly in community setting. | Need to embrace informal & formal clinical supervision- allowing more organic forms of clinical supervision will create flexibility for practitioners to adapt clinical supervision as suited to their clinical environments | Research (Pilot Implementation Evaluation Study) |
| Wright | 2012 | N/A | Nursing- generic | N/A | Review evidence base of Clinical Supervision | Paper concludes that clinical supervision was implemented based on a weak evidence base. there is little evidence that it benefits patient care and the benefits for staff and students may be outweighed by the financial and emotional costs. |  | Literature Review |

**Table 4: Literature Review Question 3 - Skills Nurses Need to Undertake Clinical Supervision**

| Author/s | Year Published | Country / Region | Nursing Discipline | Number of Participants | Aim/s | Skills Required/Training | Type of Paper |
| --- | --- | --- | --- | --- | --- | --- | --- |
| Bush | 2005 | N/A | Nurses- generic | N/A | To discuss the barriers and the reasons for them, and overcoming them | Supervisors who inspire and facilitate critical reflective practice are needed | Discussion Paper |
| Chambers & Long | 1995 | N/A | N/A | N/A | Address some of the scholarly themes and concepts involved in the experience and management of supportive clinical supervision and to stimulate future dialogue and further discussion | The supervisor is empathetic and embraces the frame of reference of the supervisee and follows his or her personal meanings. o Supervisors must have experienced supportive clinical supervision and reflection in some way to facilitate others – they need to be able to communicate skilfully in a wide variety of ways across and within a multitude of contexts. Also, must have had training on different facilitator styles. | Discussion Paper |
| Cheater & Hale | 2001 | Leicester England | Practice nurses | Unclear - (states all practice nurses in Leicestershire) | Evaluation of scheme - Assess the level of uptake of clinical supervision by practice nurses; identify factors which hindered/facilitated uptake; evaluate how far clinical supervision had influenced i) quality of clinical care ii) organisation of care iii) professional development | 2-day course needed | Evaluation |
| Chilvers and Ramsey | 2009 | England | Hospice | Not specified | Practicalities of setting up clinical supervision for RNs,HCAs & community associate practitioners (CAPs) across 3 hospice sites | Listening skills, group dynamic clinical supervision and group support; as well as practical exercises looking at cases and how to present them in a clinical supervision setting. | Literature Review |
| Hancox et al., | 2004 | Australia | Mental Health nursing | 63 | Evaluation of educational programme on clinical supervision | i) Enhancing theoretical knowledge; and appreciation of clinical supervision in mental health; nursing practice; (ii) Provision of skills to understand; and critique the various models of clinical supervision; (iii) Facilitation of the development of the skills; required to provide, and maximize the benefits of clinical supervision; and (iv) Facilitating an understanding of the legal issues and ethical dilemmas inherent in clinical supervision, and the development of strategies to overcome these. | Evaluation |
| Harvey et al., | 2020 | Australia | Unclear | 226 | Describes 8-day foundational programme (clinical supervision for Role development Training) & evaluation results | Role Development Training; Spurr Supervisor Training Model | Evaluation |
| Puffett & Perkins | 2017 | England | Palliative Care Nurses | 2 focus groups comprising 10 participants (6 RN, 2HCA’s engaged in clinical supervision) + (1RN & 1HCA not engaged in clinical supervision) | To investigate what influences palliative care nurses in their choice to engage in or decline clinical supervision. | Good listener, understanding and trustworthy and confidentiality. Need training and experience to be able to manage group. | Research (Focus Groups) |
| Rice et al., | 2007 | Northern Ireland | Mental Health Nursing | unclear | Explore ways to make clinical supervision available to all mental health nurses and in doing so to evaluate and improve their contribution to patient care | Supervisors must have sound clinical skills themselves, a strong knowledge base and be a practising clinical nurse (a therapist) | Research (Survey) |
| Severinsson | 1996 | Sweden | Nursing (across psychiatry, medical surgery, primary healthcare | 18 | Describe and analyse the nurse supervisors’ styles with emphasis on their actions during clinical supervision. Also explored expectation of the ideal clinical supervisor | Ability to enhance the supervisees’ self-esteem and autonomy by reflecting on essential themes in the supervision dialogue; and striving to attain the level of the ‘ideal’ supervisor. Listening and ability to be supportive | Research (Mixed Methods) |
| Severinsson & Hallberg | 1996 | Sweden | Registered nurses (psychiatric care, medical care, surgery, primary health, midwifery specialisms) | 18 | Investigate nurse supervisors' views of their supervisory styles, their personal qualities and their leadership role, and the development of these styles | Two specific styles the emotional & cognitive supervisory style. The supervisors' personal qualities were willingness, preparedness to show understanding, bringing out genuine feelings and 'confirming', the latter being the most important for validating the supervisee. Ability to be patient and sensitive to situations "what is in the air" | Research (Mixed Methods) |
| Sexton-Bradshaw | 1999 | England | Children’s nursing NICU & PICU | 10 NICU Staff (with experience of 1:1 clinical supervision); 15 PICU staff (without experience of clinical supervision. + 6 NICU nurses interviewed re process understanding. | Experience of supervision | Interpersonal and communication skills, role model | Research (Qualitative) |
| Sloan | 2008 | N/A | Mental health nursing | N/A | Aims to make the case for introducing accreditation of clinical supervisors in mental health nursing. | Introduction of accreditation of clinical supervisors in mental health nursing would increase the legitimacy of clinical supervision as a core activity provide recognition for an individual’s supervisory practice, lead to improvements in supervisor training, contribute to the development of its effectiveness | Discussion Paper |
| Sloan | 1999a | Unclear | Nursing (discipline not stated) | N/A | Understanding clinical supervision from a nursing perspective and does this by discussing the key empirical studies that have focused on identifying the outcomes of supervision and isolating the characteristic clinical supervision that make a good clinical supervisor, | Communication skills, supportive skills and specialist skills Promoting autonomy Being a competent therapist, ability to form supportive relationships and have relevant knowledge and clinical skills | Literature Review |
| Sloan | 1998 | N/A |  |  | Literature review into attributes of good clinical supervisors | Supervisee’s regard necessary skills and attributes to include- supervisors allowing supervisee to observe their clinical practice, using role play of interventions for supervisee, providing literature, encouraging use of new skills, having teaching ability. According to supervisors’ necessary skills and attributes include- giving feedback, specific ideas about treatment and interventions, being competent, promoting autonomy, creating warm & supportive relationship. | Literature Review |
| Sloan | 1999b | Unclear | Mental Health Nursing | 8 | Good characteristic clinical supervision of a clinical supervisor: a community mental health nurse perspective (the study after the review Sloan 1999a) | 10 key characteristic clinical supervision identified, the ability to form supportive relationships (empathy, trust mutual regard), having relevant knowledge/clinical skills, expressing a commitment to providing supervision, and having good listening skills were perceived by the staff nurses as important characteristic clinical supervision of their supervisor. Supervisees viewed their supervisor as a role model, someone who they felt inspired them, whom they looked up to and had a high regard for their clinical practice and knowledge base. Supervisor acknowledges his own limitations. Supervisor allowing supervisee to set agenda. Perceptive to the needs of the supervisee, clients and the team. | Research |
| Stacey et al., | 2020 | UK | Cross discipline | 266 | To explore the acceptability, feasibility, and experience of Resilience Based Clinical Supervision to support transition to practice in newly qualified nurses. | Requires a mental health qualification in order to have the skills to respond to and contain the distress or emotional disclosure shared especially in large groups with inconsistent membership. questioned the value of a focus on emotions also | Research |
| Temane et al., | 2014 | South Africa | Advanced Psychiatric Nursing | 8 | Explores and describes advanced psychiatric nurse practitioners’ ideas and needs regarding clinical supervision in private practice | Specific facilitative communication skills, including highly developed techniques of questioning, effectiveness in receiving and offering feedback & listening skills. Paper also looks at specific attributes that would support clinical supervision facilitation, such as an authoritative and assertive personality emotional intelligence, flexibility & a holistic and broad worldview. Professionally, expertise in supervision & psychotherapeutic experience and involvement in research. | Research (Qualitative) |
| Wilson | 1999 | Not specified | Nursing- field not specified | N/A | Author reflects on the experience of being a supervisor and outlines practicalities for the supervisor | Mentorship skills, Reflection Communication counselling, Teaching, Helicopter ability, ability to cultivate an appropriate atmosphere for disclosure, extended comm skills- he full range of communication skills, verbal and non-verbal needs to be used. Listening, attending, challenging, giving feedback and providing vision are all important. | Discussion Paper (Reflection) |
| Cutcliffe and Proctor | 1998 | UK | N/A | N/A | Discussion paper focussing on the issues of training in clinical supervision (Part 1) | Train nurses to become supervisees not supervisors. Supervisee training could commence following the first year of the common foundation programme component of diploma and undergraduate nurse education. | Discussion Paper (part of a series) |
